# The application of multiple reaction monitoring and multi-analyte profiling to HDL proteins

**DOI:** 10.1186/1476-511X-13-8

**Published:** 2014-01-08

**Authors:** Hussein N Yassine, Angela M Jackson, Chad R Borges, Dean Billheimer, Hyunwook Koh, Derek Smith, Peter Reaven, Serrine S Lau, Christoph H Borchers

**Affiliations:** 1 Department of Medicine, University of Southern California, Los Angeles, CA, USA; 2 University of Victoria - Genome British Columbia Proteomics Centre, Victoria, BC, Canada; 3 Arizona State University, Tempe, AZ, USA; 4 Statistics Consulting Laboratory, University of Arizona, Tucson, AZ, USA; 5 Phoenix VA Health Care System, Phoenix, AZ, USA; 6 Southwest Environmental Health Sciences Center, Department of Pharmacology and Toxicology, College of Pharmacy, University of Arizona, Tucson, AZ, USA; 7 Department of Biochemistry and Microbiology, University of Victoria, Victoria, BC, Canada

**Keywords:** High density lipoprotein, Proteomics, Multiple reaction monitoring, Multi-analyte panel, Diabetes, Cardiovascular disease

## Abstract

**Background:**

HDL carries a rich protein cargo and examining HDL protein composition promises to improve our understanding of its functions. Conventional mass spectrometry methods can be lengthy and difficult to extend to large populations. In addition, without prior enrichment of the sample, the ability of these methods to detect low abundance proteins is limited. Our objective was to develop a high-throughput approach to examine HDL protein composition applicable to diabetes and cardiovascular disease (CVD).

**Methods:**

We optimized two multiplexed assays to examine HDL proteins using a quantitative immunoassay (Multi-Analyte Profiling- MAP) and mass spectrometric-based quantitative proteomics (Multiple Reaction Monitoring-MRM). We screened HDL proteins using human xMAP (90 protein panel) and MRM (56 protein panel). We extended the application of these two methods to HDL isolated from a group of participants with diabetes and prior cardiovascular events and a group of non-diabetic controls.

**Results:**

We were able to quantitate 69 HDL proteins using MAP and 32 proteins using MRM. For several common proteins, the use of MRM and MAP was highly correlated (p < 0.01). Using MAP, several low abundance proteins implicated in atherosclerosis and inflammation were found on HDL. On the other hand, MRM allowed the examination of several HDL proteins not available by MAP.

**Conclusions:**

MAP and MRM offer a sensitive and high-throughput approach to examine changes in HDL proteins in diabetes and CVD. This approach can be used to measure the presented HDL proteins in large clinical studies.

## Introduction

Recent findings suggest that HDL carries over 80 proteins involved in lipid metabolism, complement regulation, acute phase response and proteinase inhibition [1]. This protein cargo appears to be remodeled in metabolic syndrome [2], cardiovascular disease [1,3] and after cholesterol therapies [4]. However, measurement of proteins associated with lipids and that span several orders of magnitude in abundance can be challenging. Conventional LC-MS/MS assays and label-free quantitation methods using spectral counting or extracted ion chromatograms (XIC) used in the previous studies [1-4] can be lengthy and are limited to small sample sizes. Multiple Reaction Monitoring (MRM), on the other hand, is a tandem MS (MS/MS) scan mode unique to triple quadrupole MS instrumentation that is capable of rapid, sensitive, and specific quantitation of peptides in highly complex sample matrices, such as plasma [5,6]. MRM is a targeted approach that requires knowledge of the molecular weight the peptide of interest and its fragmentation pattern, leading to the generation of target “transitions” for monitoring protein levels. When coupled with stable isotope peptide standards (SIS peptides), quantitation using MRM can be highly reproducible [7]. MRM quantitation has been successfully applied to plasma proteins [5,6] and, more recently, to a limited subset of HDL proteins [8]. Without pre analytical depletion or enrichment, MRM sensitivity can be compromised by the existence of high abundance proteins. Although the sensitivity of MRM allows analyses at levels down to one ng/mL, the accuracy at the lower end of the abundance range can be problematic. In this case, the use of methods that deplete the higher abundance proteins is often needed. These methods, however, can lead to the unintentional removal of proteins that are attached to the depleted proteins. An alternative approach is the use of immunoassays that offer much improved sensitivity. One example of these assays is multiplexed immunoassay panels (MAP). MAP compliments MRM at this low end and helps ensure that proteins are fully and correctly quantitated at the low range.

There has been considerable interest in understanding HDL functions in light of the strong inverse association of HDL cholesterol and cardiovascular disease (CVD) and the lack of improved CVD outcomes after raising HDL’s cholesterol content in three recent trials [9-11]. Knowledge of HDL protein composition promises to improve our understanding of HDL’s multiple functions in CVD. In this study, we optimized two approaches to measure HDL proteins using multiplexed immunoassays (Multi-Analyte Profiling-MAP) and quantitative proteomics (MRM) platforms. We extended the application of these two techniques to HDL isolated from participants with and without diabetes and CVD. Our findings suggest that MAP can be used to monitor low abundance proteins on HDL, whereas MRM allows examining higher abundance HDL proteins without the need for pre-existing antibodies.

## Materials and methods

### Clinical samples

The study was approved by the University of Arizona Institutional Review Board, and all patients provided written informed consent prior to testing. The samples were collected at University of Arizona Medicine Clinics. Participants reported to the Center for Clinical and Translational Sciences (CaTS) after an overnight fast. Samples were collected in EDTA containing tubes. The presence of cardiovascular disease was defined by a prior history of coronary artery bypass surgery (CABG), percutaneous transluminal angioplasty (PTCA), prior MI, or thrombotic stroke. Patients were older than 18 years of age. New diagnosis of diabetes was based on 2 hour oral glucose tolerance test (OGTT) > 200 mg/dl or glycated hemoglobin (HbA1c > 6.5%). Established diabetes was defined by clinical history. The study included disease free participants with advanced kidney disease, diabetes and CVD. The study excluded subjects if they met any of the following criteria: had type had 1 diabetes, were on an active weight loss program, history of cancer, HIV, or steroid use.

### HDL Isolation technique

HDL isolation by centrifugation was based on a modification of a previously published protocol [1]. In brief, potassium bromide (KBr ~55 mg) was added to 310 μl of plasma samples to create a density of 1.21 g/mL. The sample was overlaid with 200 μL of 1.21 g/mL density solution for a total volume was 500 μL. Samples were then spun at 120,000 rpm, at 16°C for 2 hours (Beckman TLX ultracentrifuge with a type 120.1 fixed angle rotor using thick-walled 500 μL Polycarbonate tubes, item 343776). The upper 125 μL solution that had a density of less than 1.21 g/mL was removed and 150 μL of NaCl/EDTA solution (0.9% (w/v) NaCl, 0.1% (w/v) EDTA, pH 7.4) was added to each tube for a final density of 1.063 g/mL. Subsequently, 225 μL of 1.06 KBr solutions in NaCl/EDTA was underlaid with a final volume of 500 μL for a second 2 hour spin at the same parameters listed. The bottom 125 μL (HDL fraction) of solution was removed for further analysis. Four HDL samples were sent to Myriad RBM to externally validate our measurements using an immunoassay in a CLIA certified laboratory. To confirm depletion of albumin and apoB proteins from HDL samples, HDL from a CVD pool was isolated with a second approach that involved long centrifugations at two sequential spins each of 10 hours duration using the above technique. Albumin levels were then measured using a commercial ELISA (Assaypro).

### Measurements of HDL proteins

We screened HDL using the human MAP panel (90 proteins) and MRM panel (56 proteins). The proteins that were detected on HDL are summarized in Table 1. Four HDL samples were used to compare protein measures by MAP and MRM. Subsequently, two pools of HDL samples (each pool 500 μL combined from 10 HDL isolates) - one pooled from ten non-diabetic subjects defined as the control group and a second pooled from ten subjects with both diabetes and CVD defined as the disease group were run on the MAP platform using the HumanMAP panel and by MRM.

**Table 1:** Proteins that were quantified on HDL from the healthy and diseased sample pool

**MAP (69 proteins quantified out of 90)**		**MRM (32 proteins quantified out of 56)**	
**Protein**	**Uniprot ID**	**ID**	**Uniprot ID**
**C-Reactive Protein (CRP)**	P02741	Apolipoprotein D	P05090
**von Willebrand Factor (vWF)**	P04275	Apolipoprotein A-I	P02647
**Adiponectin**	Q15848	Apolipoprotein A-II precursor	P02652
**Fibrinogen**	P02671, P02675, P02679	Apolipoprotein A-IV	P06727
**Serum Amyloid P-Component (SAP)**	P02743	Apolipoprotein B-100	P04114
**Sex Hormone-Binding Globulin (SHBG)**	P04278	Apolipoprotein C-I lipoprotein	P02654
**Immunoglobulin A (IgA)**	P44969	Apolipoprotein C-III	P02656
**Complement C3 (C3)**	P01024	Apolipoprotein E	P02649
**Protein S**	P26447, P06703	Apolipoprotein L1	O14791
**Thrombospondin-1**	P07996	Beta-2-glycoprotein I_Apo H	P02749
**Haptoglobin**	P00738	Apolipoprotein C-II	P02655
**Complement factor H**	P08603	Apolipoprotein(a)	P08519
**Ferritin (FRTN)**	P02794, P02792	Apolipoprotein M	O95445
**Alpha-2-Macroglobulin (A2Macro)**	P01023	PON 1	P27169
**Myeloperoxidase (MPO)**	P05164	Albumin	P02768
**Plasminogen Activator Inhibitor 1 (PAI-1)**	P05121	Alpha-1-Anti-trypsin	P01009
**Tumor Necrosis Factor Receptor 2 (TNFR2)**	P20333	Alpha-1B-glycoprotein	P04217
**Insulin**	P01308	Alpha-2-antiplasmin	P08697
**Vitronectin**	P04004	Alpha-2-HS-glycoprotein	P02765
**Calcitonin**	P01258	Clusterin	P10909
**Beta-2-Microglobulin (B2M)**	P61769	Complement C1 inactivator	P05155
**CD5L**	O43866	Complement C3	P01024
**Intercellular Adhesion Molecule 1 (ICAM-1)**	P05362	Complement C4 beta chain	P0C0L5
**CD 40 antigen (CD40)**	Q6P2H9	Complement C4 gamma chain	P0C0L5
**Carcinoembryonic Antigen (CEA)**	P06731	Complement C9	P02748
**Vascular Endothelial Growth Factor (VEGF)**	P15692	Complement factor H	P08603
**Thyroxine-Binding Globulin (TBG)**	P05543	Fibrinogen alpha chain	P02671
**Vascular Cell Adhesion Molecule-1 (VCAM-1)**	P19320	Fibrinogen beta chain	P02675
**Thyroid-Stimulating Hormone (TSH)**	P01215, P01222	Fibrinogen gamma chain	P02679
**Brain-Derived Neurotrophic Factor (BDNF)**	P23560	Fibrinopeptide A	P02671
**Matrix Metalloproteinase-3 (MMP-3)**	P08254	Haptoglobin beta chain	P00738
**Fatty Acid-Binding Protein, heart (FABP, heart)**	P05413	Hemopexin	P02790
**Tissue Inhibitor of Metalloproteinases 1 (TIMP-1)**	P01033	Heparin cofactor II	P05546
**Myoglobin**	P02144	Kininogen-1	P01042
**Immunoglobulin M (IGM)**	P01871	L-selectin	P14151
**Interleukin-8 (IL-8)**	P10145		
**Interleukin-1 beta (IL-1 beta)**	P01584		
**EN-RAGE**	P80511		
**Interleukin-1 alpha (IL-1 alpha)**	P01583		
**Monocyte Chemotactic Protein 1 (MCP-1)**	P13500		
**Macrophage Inflammatory Protein-1 beta (MIP-1 beta)**	P13236		
**Alpha-1-Antitrypsin (AAT)**	P01009		
**Fetuin A (alpha HS-glycoprotein)**	P02765		
**Interleukin-15 (IL-15)**	P40933		
**Epidermal Growth Factor (EGF)**	P01133		
**Apolipoprotein D (Apo D)**	P05090		
**Eotaxin-1**	P51671		
**Apolipoprotein(a) (Lp(a))**	P08519		
**Macrophage-Derived Chemokine (MDC)**	O00626		
**Clusterin (CLU)**	P10909		
**Prostate-Specific Antigen, Free (PSA-f)**	P07288		
**Apolipoprotein A-I (Apo A-I)**	P02647		
**Leptin**	P41159		
**Matrix Metalloproteinase-9 (MMP-9)**	P14780		
**Interleukin-10 (IL-10)**	P22301		
**Interleukin-18 (IL-18)**	Q14116		
**Interleukin-2 (IL-2)**	P60568		
**Lymphotactin**	P47992		
**T-Cell-Specific Protein RANTES (RANTES)**	P13501		
**Erythropoietin (EPO)**	P01588		
**Serum Glutamic Oxaloacetic Transaminase (SGOT)**	P17174		
**Apolipoprotein E (Apo E)**	P02649		
**Epithelial-Derived Neutrophil-Activating Protein 78 (ENA-78)**	P42830		
**Stem Cell Factor (SCF)**	P21583		
**Interleukin-13 (IL-13)**	P35225		
**Apolipoprotein B (Apo B)**	P04114		
**Growth Hormone (GH)**	P01241		
**Interleukin-4 (IL-4)**	P05112		
**Interleukin-1 receptor antagonist (IL-1ra)**	P18510		

### MAP

The samples were analyzed at Myriad RBM that uses Luminex xMAP. Luminex xMAP is a well-established particle array system that is based on beads with unique fluorescent signatures with proprietary multi-analyte panel targets assessed for cross-reactivity. This technology has been used for the detection of numerous targets, such as cytokines [12], cancer markers [13], and indicators for various disease states [14]. The assay details of this platform are well described in Myriad RBM website (http://www.myriadrbm.com). Here we applied MAP to HDL samples obtained by ultracentrifugation in both 2X2 and 10X10 isolation techniques, calculating the limit of detection and the recovery analysis after 12 dilutions. The data is presented in Additional file 1: Table S1. MAP was then applied to the control and disease HDL sample pool.

### MRM

The samples were analyzed at the University of Victoria - Genome BC Proteomics Centre with a dedicated core service for MRMs with the capacity of high throughput stable isotope peptide production for absolute quantification. We screened HDL protein using existing published transitions that were previously applied to plasma proteins [6] or to newly developed transitions from proteins that are associated with HDL (LCAT, CETP, PLTP, PON1, Apolipoprotein D, Apolipoprotein M, Apolipoprotein L1, Apolipoprotein CII) or of interest to CVD (Macrophage migration inhibitory factor, Matrix Gla protein). The transition list used is provided in Additional file 1: Table S2. For the generation of CV data, the samples were injected four times per method, with two methods in total. The total number of transitions per sample was 142 (method 1-low abundant specific targets) + 88 (method 2-general high abundance plasma protein targets). There were between 1–5 transitions monitored for each peptide. Proteins with new transitions, or those that were low in abundance were monitored using either multiple peptides (as CETP, MIF, PON1) or up to 5 transitions per peptide. The methods were not scheduled as the retention times shifted between HDL isolation procedures that can result in non-quantifiable data. Based on these replicate runs, we selected one transition for quantitation analysis based on the lowest coefficient of variation by the 4 replicate “technical” runs. These transitions are summarized in Table 2. The selection process of these transitions (to exclude interferences or amino acid modifications such as phosphorylation and glycosylation) was previously described [6,15] in detail and included in the (Additional file 1: MRM methods). Representative chromatograms of the healthy pool HDL transitions and disease pool HDL transitions are also included in the figure Additional file 1: Figure S1.

**Table 2:** HDL peptides selected for MRM

**Mass info (Q1/Q3)**	**Protein**	**Peptide sequence**	**Fragment ion**	**%CV (n = 8)**
575.3/937.5	Albumin	LVNEVTEFAK	y8	5.3
555.8/797.4	Alpha-1-Anti-trypsin	LSITGTYDLK	y7	23.9
656.8/771.4	Alpha-2-antiplasmin	LGNQEPGGQTALK	y8	116.2
399.5/490.3	Alpha-2-HS-glycoprotein	HTLNQIDEVK	y4	33.6
437.2/540.3	Antithrombin-III	DDLYVSDAFHK	y++9	63.2
409.3/599.4	Apolipoprotein M	AFLLTPR	y5	6.4
405.9/572.8	Apolipoprotein A-I	ATEHLSTLSEK	y10++	1.2
486.8/443.2	Apolipoprotein A-II precursor	SPELQAEAK	y++8	4.5
524.3/450.8	Apolipoprotein B-100	FPEVDVLTK	y++8	20.7
516.8/466.2	Apolipoprotein C-I lipoprotein	TPDVSSALDK	y++9	5.4
519.3/666.3	Apolipoprotein C-II	TAAQNLYEK	y5	3.4
598.8/244.1	Apolipoprotein C-III	GWVTDGFSSLK	b2	14.2
436.3/659.3	Apolipoprotein D	VLNQELR	y5	6.5
484.8/588.3	Apolipoprotein E	LGPLVEQGR	y5	2.1
815.9/651.3	Apolipoprotein L1	VTEPISAESGEQVER	y++12	26.7
786.5/535.3	Apolipoprotein (a)	LFLEPTQADIALLK	y++10	45.1
511.8/751.4	Beta-2-glycoprotein I_Apo H	ATVVYQGER	y6	42.6
644.8/602.3	Clusterin	ELDESLQVAER	y5	45.6
501.8/731.4	Complement C3	TGLQEVEVK	y6	50.8
557.8/629.4	Complement C4 beta chain	VDGTLNLNLR	y5	89.9
362.9/487.3	Complement C4 gamma chain	ITQVLHFTK	y++8	16.1
508.6/494.3	Complement C9	TEHYEEQIEAFK	y4	44.3
671.4/830.4	Complement factor H	SPDVINGSPISQK	y8	63.0
570.8/867.5	Fibrinogen alpha chain	GSESGIFTNTK	y8	50.1
497.9/600.3	Fibrinogen gamma chain	YEASILTHDSSIR	y++11	17.3
768.8/1077.5	Fibrinopeptide A	ADSGEGDFLAEGGGVR	y11	95.1
490.8/562.3	Haptoglobin beta chain	VGYVSGWGR	y5	59.9
610.8/480.3	Hemopexin	NFPSPVDAAFR	y++9	147.9
514.8/814.4	Heparin cofactor II	TLEAQLTPR	y7	31.9
626.3/1051.5	Kininogen-1	TVGSDTFYSFK	y9	28.9
497.8/794.4	L-selectin	AEIEYLEK	y6	64.4
592.8/943.5	PON 1	IQNILTEEPK	y8	51.0

HDL proteins that were quantified using MRM. The CV was calculated using 4 technical runs.

### Statistical analysis

We used the statistical program R2.1 (R core development team). The characteristics of the two pools were compared using an independent sample test or a proportion test. The correlation coefficient and the p value generated between plasma proteins and HDL proteins was obtained using spearman correlation test.

## Results and discussion

### Description of study participants

The goal of this project was to use sensitive and high throughout approach to analyze HDL proteins in health and vascular disease such as diabetes and CVD. The samples were selected to detect differences in HDL proteins between healthy controls and patients with chronic vascular disease. The study demographics and biochemical measurements are listed in Table 3. The two groups were age and sex matched. All diseased individuals had a history of CVD events prior to participation. As shown in Table 3, diseased subjects were obese, with lower HDL cholesterol, elevated triglyceride levels, uncontrolled diabetes, elevated inflammation (CRP), and evidence of chronic kidney disease as revealed by the elevated plasma creatinine.

**Table 3:** Demographic and biochemical characteristics of study participants

	**Control (n = 10)**	**Disease (n = 10)**	**p value**
Age (yrs)	58.4 (6.1)	62.2 (7.5)	0.233
Sex ( M:F)	4:6	5:5	0.99
BMI (kg/ m^2^)	24.3 (4.5)	37.8 (7.7)	0.001
Systolic BP (mm Hg)	122.4 (9.4)	131.4 (17.4)	0.173
Diastolic BP (mmHg)	74.8 (5.7)	70.7 (9.8)	0.275
LDL (mg/dL)	138.5 (35)	114.1 (42.2)	0.178
HDL (mg/dL)	58.7 (9.5)	36.1 (7.46)	<0.001
CRP (mg/dL)	2.6 (2.27)	13.3 (11)	0.014
Triglycerides ( mg/dL)	111.1 (55)	309.2 (205)	0.014
Creatinine (mg/dL)	0.7 (0.15)	1.65 (0.93)	0.011
Glycated Hemoglobin %	5.4 (0.38)	9.2 (2.71)	0.002
Urine Microalbumin (mcg/mg creatinine)	8.4 (6.7)	1667.5 (2596)	0.114

The samples were pooled from the control and disease groups. Values are means (SD).

### HDL and Plasma proteins

**Figure 1:**
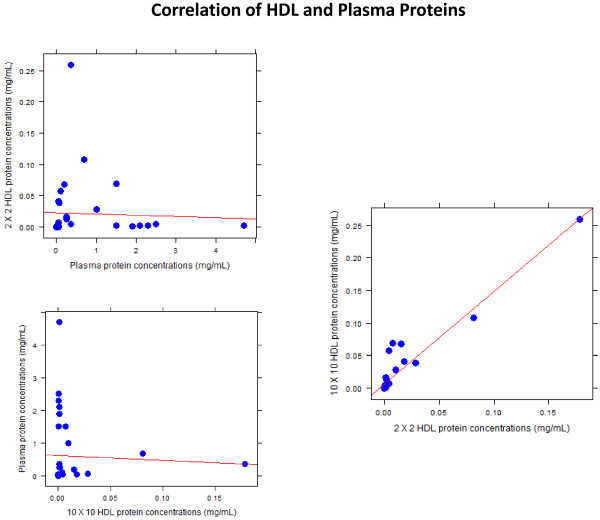
**Correlation of Plasma and HDL proteins using MAP.** Plasma and HDL proteins were correlated using MAP. HDL (2×2) and HDL (10×10) represent two different sequential centrifugation techniques that were 2 or 10 hours duration at the upper and lower densities of HDL. As shown in the figure, there was no correlation between the concentrations of proteins between plasma and HDL. In contrast, the concentration of proteins isolated using 2.2 and 10.10 were highly correlated (r = 0.95, P < 0.001).

One challenge in HDL proteomics is differentiating whether proteins are actually present within HDL or are predominately carried within plasma and have become loosely associated with HDL before or during the isolation procedure. We initially isolated HDL using a two sequential spins (2 hrs × 2) and we are able to detect several low abundance proteins commonly associated with atherogenesis and inflammation using MAP (Table 1) of participants with both diabetes and CVD. To confirm whether these proteins are part of the HDL fraction or loosely attached plasma proteins, a longer centrifugation process was employed to isolate HDL (10 hrs × 2). Plasma albumin levels were determined to be 45 mg/mL. After the (2 hrs × 2) centrifugation, albumin levels within the HDL fraction were 0.1 mg/mL. After prolonged sequential centrifugation step (10 hrs × 2), albumin levels were below the detection limits of this assay. Proteins detectable on HDL (by both centrifugation techniques) assessed by MAP, along with plasma protein concentrations are summarized in Table 4. We then correlated the concentrations of these proteins between the different fractions. We found no correlation between plasma and HDL proteins identified in either the (2 hrs ×2) or (10 hrs ×2) HDL isolation preparations. For example, Apo A-I concentrations of 0.36, 0.27, 0.18 mg/mL in plasma HDL (2 hrs × 2) and HDL (10 hrs × 2), respectively shows clear retention on HDL compared to fibrinogen with concentrations of 4.7, 0.0021, 0.0012 mg/mL. These findings confirm that non-HDL proteins were efficiently depleted with the longer centrifugation. In contrast, the HDL proteins isolated with the (2 hrsx2) and (10 hrs × 10) methods were highly correlated (Figure 1, r = 0.95, p < 0.001). As expected the concentrations of HDL proteins were greater in the (2 hrs × 2) than the (10 hrs × 2) technique (Table 3). The concentration of Apo A-I in plasma was low in this sample pool from diseased participants compared to healthy controls. Published Apo A -I levels are in the 0.9-1.6 mg/mL range. This may suggest significant HDL remodeling in advanced vascular disease.

**Table 4:** **Concentrations of Plasma and HDL proteins from the pooled sample of the diseased subject (mg/mL)**

**Protein**	**Plasma**	**HDL 2X2**	**HDL 10X10**
Apolipoprotein A-I (Apo A-I)	0.36	0.259	0.178
Alpha-1-Antitrypsin (AAT)	1.5	0.0688	0.00736
Immunoglobulin A (IgA)	2.5	0.00422	0.00054
Immunoglobulin M (IGM)	2.1	0.00275	0.00117
Complement C3 (C3)	1.5	0.00211	0.000792
Fibrinogen	4.7	0.00209	0.00118
Haptoglobin	2.3	0.00191	0.000438
Alpha-2-Macroglobulin	1.9	0.000857	0.00104
Apolipoprotein (a) (Lp (a))	0.684	0.108	0.081
Fetuin-A	0.2	0.068	0.015
Complement Factor H	0.1	0.057	0.0036
Apolipoprotein D (Apo D)	0.05	0.041	0.018
Apolipoprotein C-III (Apo C-III)	0.07	0.039	0.028
Apolipoprotein B (Apo B)	1.0	0.028	0.01
Apolipoprotein H (Apo H)	0.246	0.016	0.00091
Vitronectin	0.26	0.013	0.0018
Apolipoprotein E (Apo E)	0.04	0.0074	0.0042
Clusterin (CLU)	0.37	0.0042	0.0011
Thyroxine-Binding Globulin (TBG)	0.044	0.0025	0.00033
Beta-2-Microglobulin	0.0041	0.00094	0.00033
Vitamin K-Dependent Protein S	0.05	0.00027	0.000074
Serum Amyloid P-Component	0.017	0.00015	0.000051
C-Reactive Protein (CRP)	0.015	0.00013	0.000023
CD5 (CD5L)	0.00053	0.000106	0.0000096
Vascular Cell Adhesion Molecule-1 (VCAM-1)	0.000761	0.000039	0.000011
Myoglobin	0.00013	0.000031	0.000016
Tissue Inhibitor of Metalloproteinases 1 (TIMP-1)	0.000104	0.0000097	0.0000012
Thrombospondin-1	0.00482	0.000008	0.0000036
Plasminogen Activator Inhibitor 1 (PAI-1)	0.000072	0.000004	0.0000005
Tumor Necrosis Factor Receptor 2 (TNFR2)	0.000014	0.0000017	0.0000001
T-Cell-Specific Protein RANTES (RANTES)	0.0000097	0.0000015	0.0000004
EN-RAGE	0.000067	0.0000001	< LOW >
Myeloperoxidase (MPO)	0.00183	< LOW >	0.000019
Sex Hormone-Binding Globulin (SHBG)	0.0016195	0.0000218	0.0000006

HDL 2.2 and 10.10 refer to two sequential ultra-centrifugations 2 or 10 hours each. Samples were diluted 12 times and were run once at each dilution. The reported concentrations were in the linear range of the assay (Additional file 1: Table S1). The strength of this technique is in the ability to measure these HDL proteins across a wide concentration range.

### Correlation of MAP and MRM

MRM’s performance for low abundance proteins is limited without prior enrichment. Thus, MAP allows the measurements of low abundance proteins on HDL. There were 10 proteins common to both MAP and MRM, however, one protein (Apo E) was not detected in our samples in 3 out of 4 samples by analyzed by MRM. We correlated 9 proteins measured by both approaches in 4 HDL samples. The results are summarized in Table 5. We did not observe a good correlation for lipoprotein (a), Apo D and Apo CIII between the two methods. Most of Lipoprotein (a) is associated with LDL and present in low abundance on HDL [16] reflecting a challenge for measuring this protein on HDL by MRM. Apo D on the other hand, has a high degree of homology to retinol binding protein or other members of the alpha 2 microglobulin protein superfamily [17]. This might potentially pose a challenge for the MAP technique. Apo CIII was another protein that was measured by MAP and MRM with a weak correlation between the two measurement platforms. It is not clear why these assays were discordant. It is possible that the peptide used is not a good surrogate of Apo CIII levels. Given that Apo CIII exists in multiple isoforms [18], both assays could be measuring different forms.

**Table 5:** **Correlation between MRM and MAP **(**Spearman**)

**Protein**	**R**	**P value**
Apolipoprotein A-I (Apo A-I)	0.99	0.001
Fetuin-A	0.97	0.03
Apolipoprotein H (Apo H)	1	0.001
Clusterin (CLU)	1	0.008
Apo B	0.99	0.002
Lipoprotein (a)	0.8	0.2
Complement factor H	0.99	0.001
Apo D	-0.2	0.5
Apo CIII	- 0.06	0.9

The correlation between Myriad and MRM was based on the concentration of common protein targets from the 4 HDL samples. There were 10 common proteins between MAP and MRM. Apo E was not detectable in 3 out of the 4 samples by MRM and thus was not included in this list. For most of the common proteins, the two assays were highly correlated.

### HDL Proteome in diabetes and CVD

**Figure 2:**
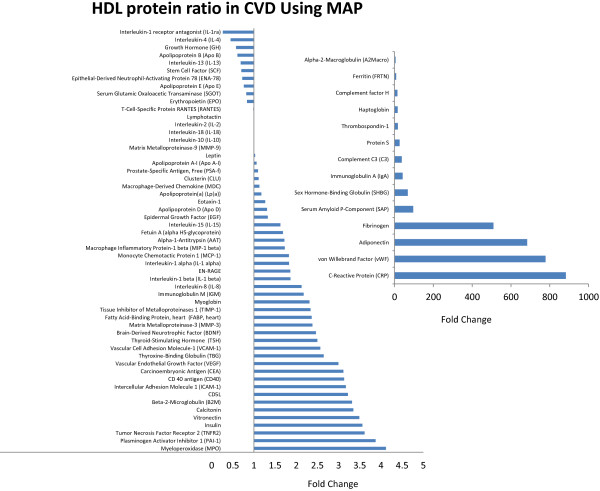
**HDL protein ratio in disease vs healthy using MAP.** Two pooled HDL samples of 10 non-diabetic participants and 10 subjects with diabetes and CVD were submitted to proteins analysis by MAP. The figure shows the ratio of HDL proteins in the diseased and the control individuals. Several proteins implicated in atherogenesis (MPO, TNRF 2, IL1 beta) were detected in the HDL of diseased individuals.

**Figure 3:**
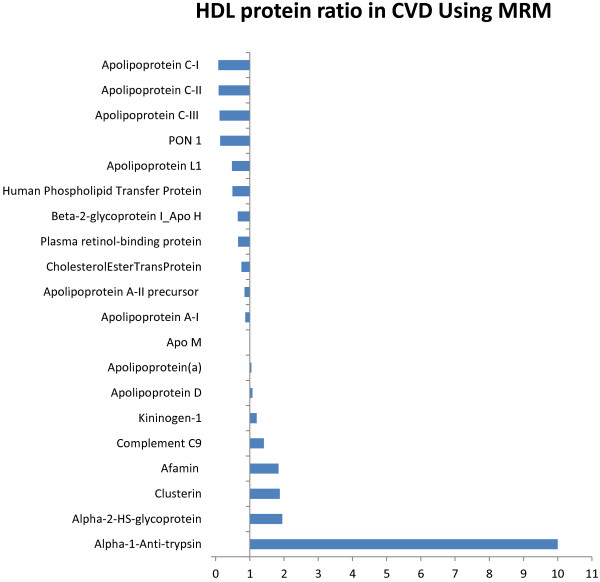
**HDL protein ratio in disease vs healthy using MRM.** Two pooled HDL samples of 10 non-diabetic participants and 10 subjects with diabetes and CVD were submitted to proteins analysis by MRM. The figure shows the ratio of HDL proteins in the diseased and the control individuals using MRM. Proteins involved in lipid metabolism were decreased whereas proteins involved in inflammation were increased on HDL of participants with CVD.

There is strong inverse association of HDL cholesterol and cardiovascular disease (CVD). However, recent studies suggested that niacin or CETP inhibition designed to raise HDL-C content did not lead to improved CVD outcomes [9-11]. This discrepancy highlights the need to revise our view of HDL and its functions. One previous study demonstrated that HDL acquires an inflammatory phenotype in acute coronary syndrome [3]. Our results suggest that examining HDL by MAP and MRM can reveal important insights into its biology. One significant finding in our study is the ability to detect for the first time important proposed mediators of atherosclerosis (ENRAGE, MPO, and PAI-1) on HDL using MAP. We also optimized an MRM transition library for the high throughput monitoring of HDL peptides. To investigate the HDL proteome in CVD, HDL-containing samples from 10 non-diabetic participants and HDL from subjects with both diabetes and prior CVD events were pooled into a control and a DM/CVD sample pool. 69 out of the 90 humanMAP proteins showed acceptable recovery in response to multiple dilutions (Additional file 1: Table S1) and were above the lower limit of detection in both sample pools. Figure 2 represents the ratio of the 69 proteins on HDL between disease and control participants. Our findings suggested that several atherosclerosis-associated and inflammatory proteins were increased on HDL of diseased individuals. In addition, MPO, PAI-1, IL1beta, and ENRAGE were only detected on HDL of diseased individuals. We acknowledge that by pooling the samples, the clinical utility of this approach is limited. A larger study of subjects with and without disease is needed to fully characterize the distribution of these low abundance proteins on HDL. Unlike MAP, the sensitivity of MRM is limited without prior sample enrichment. Hence, the accuracy of the assay is compromised when sample concentrations fall below the linear range of the assay as evidenced by the increased coefficient of variation (CV) in several MRM proteins. By MRM, we targeted transitions of 56 proteins. We were able to detect 32 proteins of which 11 proteins had CVs less than 20% on the replicate runs (Tables 1 and 2). Clusterin is a high abundant protein that is associated with HDL. We were able to quantitate clusterin accurately in plasma (CV < 5% [6]); however we were not able to reliably measure it in HDL as was previously demonstrated [8] reflecting the challenges of using MRMs for protein quantification when levels of a given target falls out of the dynamic range in diseased states. Comparing HDL proteins in the CVD versus control pool by MRM revealed the depletion of several lipid metabolism proteins such as Apo CI, CII and PON-1 in CVD. In contrast, the concentrations of several acute phase proteins such as clusterin, Complement C9, alpha-1-antitrypsin were increased in CVD (Figure 3). These findings would facilitate conducting larger studies using the presented approach to examine the effect of inflammation and atherosclerosis associated proteins on HDL composition and function in individuals with diabetes and CVD.

### Challenges of measuring lipid associated proteins

Measurement of lipid associated proteins has been successful using immunoassays based on nephelometry and mass spectrometry [1,4,8]. Nephelometry is not widely available and not amenable to multiplexing. Conventional mass spectrometry techniques using spectral counting or extracted ion chromatograms can be lengthy and challenging in large sample sizes. Here, we present two alternatives (MAP and MRM) that are amenable to multiplexing, and are high throughput. MAP is limited by the availability and quality of pre-exiting antibody panels. MRM, on the other hand, can lose sensitivity at the lower end of abundance. Although the performance of MRM in lower abundance HDL proteins was inferior to immune based assays, the performance of MRM is likely to improve with the development of more sensitive mass spectrometry analyzers and better sample fractionation methods.

### Limitations

There are several limitations of this study. First, the present study was done with two pools of clinical samples. As such, the present work describes an alternative analytical tool for the measurement of HDL proteins. Application of these assays to larger clinical data sets is feasible and will allow determination of their clinical utility. Second, the digestion efficiency of each protein monitored by MRM was not assessed. However, we have previously shown [19] that if the digestion procedure is consistent and reproducible, then the ability to compare quantitative values between samples is maintained. Thus, the results presented are better described as “relative accurate abundances”. Despite this limitation, several peptides quantitated by MRM were correlated with measurement using MAP (Table 5) affirming our claim that the MRM assay can provide absolute quantitation. A third limitation of this study is that we did not use an independent method of HDL isolation (such as reciprocal co-immunoprecipitation of a given protein with Apo A-I, or gel filtration as suggested by Davidson et al. [20]) to validate that presence of these low abundance proteins on HDL. However, HDL isolated by a longer centrifugation step had successfully depleted albumin with several of the low abundance proteins still detectable on HDL. In addition, the proteins on HDL and the corresponding plasma concentrations did not correlate, whereas the two HDL fractions were highly correlated. This finding suggests that these low abundance proteins were not contaminant plasma proteins. Our findings however, need to be replicated in a larger study group.

## Conclusions

This study suggests the feasibility of measuring HDL proteins using MRM and MAP. The application of MAP and MRM to the HDL proteome offers the potential to improve our understanding of HDL functions and help direct interventions aimed at remodeling the HDL phenotype in diabetes and CVD.

## Abbreviations

HDL: High density lipoprotein; CVD: Cardiovascular disease; Apo A-I: Apolipoprotein A1; MRM: Multiple reaction monitoring; MAP: Multi-analyte profiling.

## Competing interests

The author’s declare that they have no competing interests.

## Authors’ contributions

Participated in research design: HY, SL, DS, CB, DB; conducted experiments: HY, HK, AJ; performed data analysis and interpretation: HY, DB; contributed to the writing of the manuscript: PR, CB; critically revised the manuscript: PR; all authors read and approved the final manuscript.

## Supplementary Material

Additional file 1 **lists (1) details of MRM method summary. (2) Table S1.** showing the HDL protein concentrations after 12 dilutions for determination of lower limit of detection (linearity analysis) **(3) Table S2.** listing all the transitions used to screen for HDL proteins **(4) Figure S1.** showing representative chromatograms from the 4 replicate runs. Click here for file
