# Supplementary material for: The application of multiple reaction monitoring and multi-analyte profiling to HDL proteins
Source: Lipids Health Dis. 2014 Jan 8;13:8. doi: 10.1186/1476-511X-13-8 (PMC3900256; doi:10.1186/1476-511X-13-8)

Additional File

The application of multiple reaction monitoring and multi-analyte profiling to HDL proteins

Hussein N Yassine[1*]
[*] Corresponding author
Email: hyassine@usc.edu

Angela M Jackson[2]
Email: ajackson@proteincentre.com

Chad R Borges[3]
Email: chad.borges@asu.edu

Dean Billheimer[4]
Email: dean.billheimer@arizona.edu

Hyunwook Koh[1]
Email: hyunwook.koh@usc.edu

Derek Smith[3]
Email: derek@proteincentre.com

Peter Reaven[5]
Email: peter.reaven@va.gov

Serrine S Lau[6]
Email: lau@pharmacy.arizona.edu

Christoph H Borchers[2,7]
Email: christoph@proteincentre.com

Methods:

MRM

1. Developing HDL Peptide Transitions for MRM: An HDL sample isolated using the 2X2 technique from ten random plasma sample pool was screened for transitions of 54 plasma proteins previously published [9].32 proteins were detected on HDL. For known HDL proteins for which we did not have MRM transitions (PON1, LCAT, CETP, PLTP, Apo M, and Apo F), we purchased the corresponding recombinant proteins and determined the best peptide targets by LC/MS/MS. The top peptides were chosen based on the MASCOT score (compared to the expected values), sequence (avoiding residues that could cause quantitation problems such as miscleavage), length (for SIS peptide synthesis considerations), MS/MS spectra (emphasis on strong y-ions to retain the aa mass shift in the corresponding stable-labeled SIS peptide), and peptide intensity. We synthesized the SIS peptides for the top 2 peptides for each protein.

2. Sample Preparation prior to LC/MRM-MS: Samples were first diluted by the addition of 140µL of 37.5 mM ammonium bicarbonate to each 100 µL of sample. Each diluted HDL sample was denatured by adding 30 µL of 10% w/v sodium deoxycholate (NaDOC) in 37.5mM ammonium bicarbonate. Disulphide bonds were reduced by the addition of 7.46 µL of 50 mM *tris* (2-carboxyethyl) phosphine (TCEP, in 37.5mM ammonium bicarbonate), and incubating at 60°C for 30 min in a dry-air incubator. Free sulfhydryl groups were alkylated by the addition of8.28 µL of 100 mM iodoacetamide (in 37.5mM ammonium bicarbonate), and incubating at 37°C for 30 min in a dry-air incubator. Any remaining iodoacetamide was quenched by the addition of 8.28 µL of 100 mM DTT (in 37.5mM ammonium bicarbonate)and incubation at 37°C for 30 min in a dry-air incubator. Six µL of sequencing-grade trypsin (0.4 µg/µL (Promega) in 37.5mM ammonium bicarbonate) was added to each sample. The final volume of each digest was 300 µL, and digestion was conducted at 37°C for 16 hours in a dry air incubator.

3. SIS peptide addition & solid phase extraction: Digestion was stopped by the addition of an acidified stable-isotope-labeled standard (SIS) peptide mixture in formic acid, to give a final formic acid concentration of 0.5 % v/v and to reduce the pH to <3, which inactivates trypsin and precipitates NaDOC). Samples were centrifuged for 10 min at 12,000 × *g* (23°C) to remove the NaDOC precipitate. The supernatant containing the peptides was desalted and concentrated by solid phase extraction using Waters Oasis HLB 1cc columns (10 mg). The eluted samples were frozen and lyophilized to dryness overnight. Prior to the LC/MRM-MS analysis, samples were rehydrated in a volume of Solvent A (0.1% v/v formic acid) to obtain a concentration of 0.5 µg/µl of original sample.

4. LC/MRM-MS method: The MS analyses were performed on an AB/MDS Sciex 4000 QTRAP equipped with an Eksigent NanoLC-1Dplus LC system. The trapping column used was a5 x 0.3 mm C18 Pep map column, packed with 5 µm particles (Dionex/LC Packings). The analytical column was a75 µm x 150 mm Reprospher 100 C18 Aqua column, packed with 3µm particles, 100 Å pore size, packed in-house under argon. The solvent system consisted of solvent A (100% H_2O, 0.1% v/v formic acid), and solvent B = 90% aqueous acetonitrile, 0.1% v/v formic acid, and the loading pump solvent was 2% aqueous acetonitrile, 0.1% v/v formic acid. The on-line analyses were 43 min in length and the gradient was constructed as follows: samples were loaded onto the trapping column at 10 µL/min (2% aqueous acetonitrile, 0.1% v/v formic acid) for 3 min, followed by a 2 min linear gradient from 3% to13% solvent at 300 nL/min, a10 min

linear gradient at 300 nL/min from 13% to20% solvent B, a 9 min linear gradient at 300 nL/min from 20% to 27% solvent B, and a final 6 min linear gradient at 300 nL/min from 27% to44% solvent B. A blank solvent injection was run between all samples to prevent sample carryover on the HPLC column. Each sample was analyzed twice, using a different MRM method each time. An AB/MDS Sciex 4000 QTRAP with a Michrom Captive Spray source, controlled by Analyst 1.5 software (Applied Biosystems) was used for all of the LC/MRM-MS analyses. All acquisition methods used the following instrument parameters: 1300-1500 V ion spray voltage, a 110°C interface heater temperature, an MS operating pressure of 3.5×10^{-5}Torr, and Q1 and Q3 set to unit resolution (0.6 – 0.8 Da FWHH). MRM acquisition methods were constructed using 1 or 2 ion pairs per peptide with empirically-tuned DP and CE voltages for each transition. A default collision cell exit potential of 23 V was used for all MRM ion pairs, with a target cycle time of 2 seconds.

5. MRM data acquisition: All MRM data was processed using MultiQuant 1.2 (Applied Biosystems) with the MQL algorithm for peak integration. Automatic peak detection,3-point Savitsky-Golay smoothing, a peak-splitting factor of 2, and default MultiQuant values for the noise percentage and baseline subtraction window were used. All integrated peaks were manually inspected to ensure correct peak detection and integration.

Table S1: Linearity Analysis of HDL proteins using MAP

Analytes	Adiponectin	Adiponectin		Alpha-1-Antitrypsin (AAT)	Alpha-1-Antitrypsin (AAT)		Alpha-2-Macroglobulin (A2Macro)	Alpha-2-Macroglobulin (A2Macro)		Alpha-Fetoprotein (AFP)	Alpha-Fetoprotein (AFP)		Apolipoprotein A-I (Apo A-I)	Apolipoprotein A-I (Apo A-I)		Apolipoprotein B (Apo B)	Apolipoprotein B (Apo B)		Apolipoprotein C-III (Apo C-III)
Units		ug/mL			mg/mL			mg/mL			ng/mL			mg/mL			ug/ml		
Multiplex		HMP8			HMP3			HMP8			HMP1			HMP3			HMPC46		
LLOQ		0.0018			6.5E-07			0.0026			1.1			2.3E-07			0.0034		
<u>Samples</u>	MFI	Result	Linearity %	MFI	Result	Linearity %	MFI	Result	Linearity %	MFI	Result	Linearity %	MFI	Result	Linearity %	MFI	Result	Linearity %	MFI
**10X 10 {1:2}**	QNS	QNS		QNS	QNS		QNS	QNS		QNS	QNS		QNS	QNS		QNS	QNS		QNS
**10X 10 {1:6}**	6.0	-		182	1.3E-05		5912	-		6.0	-		205	6.7E-06		4245	1.3		1400
**10X 10 {1:18}**	3.0	-	-	388	3.4E-05	13%	6682	-	-	5.0	-	-	387	1.3E-05	17%	6021	2.3	19%	2306
**10X 10 {1:54}**	4.0	-	-	649	7.9E-05	14%	6802	-	-	6.0	-	-	589	2.2E-05	21%	6338	2.5	30%	3427
**10X 10 {1:162}**	5.0	-	-	902	0.00017	16%	6875	-	-	6.0	-	-	963	3.9E-05	18%	5684	2.1	41%	3955
**10X 10 {1:486}**	5.0	-	-	766	0.00011	50%	7296	-	-	7.0	-	-	1391	6.5E-05	20%	3228	0.89	78%	4565
**10X 10 {1:1458}**	4.0	-	-	403	3.5E-05	104%	7167	-	-	5.0	-	-	1897	0.00011	19%	1170	0.30	100%	4568
**10X 10 {1:4374}**	4.0	-	-	147	1.0E-05	117%	6850	-	-	5.0	-	-	2096	-	-	459	0.12	80%	1861
**10X 10 {1:13,122}**	3.0	-	-	56	3.5E-06	97%	7042	-	-	7.0	-	-	1437	6.9E-05	-	166	0.046	89%	645
**10X 10 {1:39,366}**	4.0	-	-	25	1.3E-06	91%	7053	-	-	6.0	-	-	605	2.2E-05	103%	67	0.018	86%	161
**10X 10 {1:118,098}**	2.0	-	-	16	-	-	6723	-	-	7.0	-	-	220	7.2E-06	103%	41	0.010	58%	36
**10X 10 {1:354,294}**	3.0	-	-	9.0	-	-	7251	-	-	5.0	-	-	102	3.0E-06	80%	28	0.0067	52%	22
**2X2 {1:2}**	QNS	QNS		QNS	QNS		QNS	QNS		QNS	QNS		QNS	QNS		QNS	QNS		QNS
**2X2 {1:6}**	86	0.031		114	7.6E-06		6177	-		8.0	-		143	4.5E-06		8743	-		1432
**2X2 {1:18}**	24	0.0070	148%	196	1.4E-05	18%	6614	-	-	8.0	-	-	299	1.0E-05	15%	6924	3.1	-	2044
**2X2 {1:54}**	10	-	-	284	2.2E-05	21%	7296	-	-	8.0	-	-	536	1.9E-05	17%	5671	2.1	50%	2847
**2X2 {1:162}**	6.0	-	-	401	3.5E-05	21%	6813	-	-	7.5	-	-	830	3.2E-05	20%	3125	0.85	80%	3935
**2X2 {1:486}**	4.0	-	-	695	9.0E-05	13%	7035	-	-	8.0	-	-	1272	5.7E-05	19%	972	0.25	114%	5043
**2X2 {1:1458}**	4.0	-	-	866	0.00015	20%	7085	-	-	8.5	-	-	1699	9.1E-05	21%	374	0.10	82%	4484
**2X2 {1:4374}**	3.0	-	-	793	0.00012	41%	7518	-	-	6.0	-	-	2280	-	-	140	0.039	87%	2797
**2X2 {1:13,122}**	3.0	-	-	344	2.8E-05	141%	7271	-	-	6.0	-	-	1753	9.6E-05	-	62	0.016	79%	758
**2X2 {1:39,366}**	4.0	-	-	152	1.0E-05	90%	7051	-	-	7.0	-	-	1033	4.3E-05	75%	38	0.0096	57%	262
**2X2 {1:118,098}**	4.0	-	-	51	3.1E-06	112%	7357	-	-	7.0	-	-	380	1.3E-05	108%	29	0.0071	45%	70
**2X2 {1:354,294}**	2.0	-	-	25	1.3E-06	81%	7051	-	-	6.0	-	-	141	4.4E-06	100%	23	0.0054	44%	24

Samples were obtained from diseased sample pool. HDL was isolated by 10X10 or 2X2 centrifugation technique. MFI refers to Median Fluorescent Intensity, is based on beads with unique fluorescent signatures.The LLOQ (Lower Limit of Quantitation) is the lowest concentration of an analyte in a sample that can be reliably detected and at which the total error meets the laboratory's requirements for accuracy. In this case, the laboratory's requirement for accuracy is the concentration of an analyte at which the coefficient of variation of replicate standard samples is 30%. QNS indicates Quantity Not Sufficient for testing. Linearity % calculated as initial result divided by next dilution result multiplied by 3. Results below the LLOQ have been omitted and replaced with "-". <HIGH> results greater than the assay range have been omitted and replaced with "-".Results highlighted in blue reflect acceptable dilution linearity as defined as between 70-130% recovery. Analytes without highlighted blue results reflect either lack of detection in the samples or lack of linearity between dilutions.

Table S1: Linearity Analysis

Analytes	Apolipoprotein C-III (Apo C-III)		Apolipoprotein D (Apo D)	Apolipoprotein D (Apo D)		Apolipoprotein E (Apo E)	Apolipoprotein E (Apo E)		Apolipoprotein H (Apo H)	Apolipoprotein H (Apo H)		Apolipoprotein(a) (Lp(a))	Apolipoprotein(a) (Lp(a))		Beta-2-Microglobulin (B2M)	Beta-2-Microglobulin (B2M)		Calcitonin	Calcitonin
**Units**	ug/mL			ug/ml			ug/ml			ug/mL			ug/mL			ug/mL			pg/mL
**Multiplex**	HMP3			HMPC47			HMPC47			HMP3			HMP2			HMP8			HMP1
**LLOQ**	0.00028			0.16			0.0076			0.00010			0.0051			0.00028			13
**Samples**	**Result**	**Linearity %**	**MFI**	**Result**	**Linearity %**	**MFI**	**Result**	**Linearity %**	**MFI**	**Result**	**Linearity %**	**MFI**	**Result**	**Linearity %**	**MFI**	**Result**	**Linearity %**	**MFI**	**Result**
**10X 10 {1:2}**	QNS		QNS	QNS		QNS	QNS		QNS	QNS		QNS	QNS		QNS	QNS		QNS	QNS
**10X 10 {1:6}**	-		263	19		4282	1.2		6177	0.25		2592	1.5		2278	0.081		5.0	-
**10X 10 {1:18}**	-	-	481	5.8	107%	3393	0.94	43%	6101	0.25	34%	4730	3.0	16%	2406	0.11	24%	3.0	-
**10X 10 {1:54}**	-	-	991	2.0	96%	1541	0.41	76%	3364	0.092	89%	5936	-	-	1650	0.035	106%	5.0	-
**10X 10 {1:162}**	-	-	1912	0.83	80%	579	0.16	88%	1407	0.032	95%	4330	2.7	-	772	0.013	92%	3.5	-
**10X 10 {1:486}**	-	-	3571	0.33	85%	160	0.042	123%	463	0.011	102%	1705	0.93	97%	243	0.0041	102%	5.5	-
**10X 10 {1:1458}**	-	-	4777	0.17	65%	57	0.013	108%	161	0.0038	94%	631	0.34	90%	68	0.0015	92%	4.0	-
**10X 10 {1:4374}**	-	-	6179	-	-	23	-	-	52	0.0011	111%	188	0.11	103%	23	0.00053	95%	3.0	-
**10X 10 {1:13,122}**	0.017	-	5538	-	-	15	-	-	20	0.00033	114%	76	0.048	78%	11	-	-	1.0	-
**10X 10 {1:39,366}**	0.0043	134%	6001	-	-	15	-	-	10	-	-	26	0.015	103%	11	-	-	3.0	-
**10X 10 {1:118,098}**	0.0010	137%	5498	-	-	12	-	-	6.0	-	-	12	-	-	7.0	-	-	6.0	-
**10X 10 {1:354,294}**	0.00061	57%	5880	-	-	12	-	-	5.0	-	-	5.0	-	-	7.0	-	-	3.0	-
**2X2 {1:2}**	QNS		QNS	QNS		QNS	QNS		QNS	QNS		QNS	QNS		QNS	QNS		QNS	QNS
**2X2 {1:6}**	-		213	-		3278	0.90		3057	0.081		2822	1.6		1761	0.039		6.0	-
**2X2 {1:18}**	-	-	407	7.7	-	3994	1.1	27%	4070	0.12	22%	5290	-	-	2326	0.090	15%	3.0	-
**2X2 {1:54}**	-	-	668	3.5	73%	2750	0.75	50%	5637	0.21	19%	7387	-	-	2533	0.21	14%	3.0	-
**2X2 {1:162}**	-	-	1226	1.5	77%	1248	0.33	75%	6686	0.30	24%	5308	-	-	1580	0.032	214%	4.0	-
**2X2 {1:486}**	-	-	2384	0.62	82%	339	0.092	121%	5747	0.22	45%	2018	1.1	-	775	0.013	85%	7.0	15
**2X2 {1:1458}**	-	-	4529	0.20	104%	101	0.026	119%	2803	0.072	101%	790	0.43	87%	225	0.0039	110%	4.5	-
**2X2 {1:4374}**	-	-	5116	-	-	37	-	-	1001	0.023	107%	257	0.15	96%	81	0.0017	75%	3.0	-
**2X2 {1:13,122}**	0.021	-	5711	-	-	19	-	-	299	0.0069	109%	83	0.052	95%	21	0.00047	123%	3.0	-
**2X2 {1:39,366}**	0.0068	101%	5566	-	-	16	-	-	103	0.0024	97%	27	0.016	108%	10	-	-	3.0	-
**2X2 {1:118,098}**	0.0020	115%	5761	-	-	14	-	-	37	0.00075	105%	10	-	-	8.0	-	-	3.5	-
**2X2 {1:354,294}**	0.00068	97%	5051	-	-	13	-	-	16	0.00023	108%	7.0	-	-	7.0	-	-	2.0	-

Samples were obtained from diseased sample pool. HDL was isolated by 10X10 or 2X2 centrifugation technique. MFI refers to Median Fluorescent Intensity, is based on beads with unique fluorescent signatures.The LLOQ (Lower Limit of Quantitation) is the lowest concentration of an analyte in a sample that can be reliably detected and at which the total error meets the laboratory's requirements for accuracy. In this case, the laboratory's requirement for accuracy is the concentration of an analyte at which the coefficient of variation of replicate standard samples is 30%. QNS indicates Quantity Not Sufficient for testing. Linearity % calculated as initial result divided by next dilution result multiplied by 3. Results below the LLOQ have been omitted and replaced with "-". <HIGH> results greater than the assay range have been omitted and replaced with "-".Results highlighted in blue reflect acceptable dilution linearity as defined as between 70-130% recovery. Analytes without highlighted blue results reflect either lack of detection in the samples or lack of linearity between dilutions.

Table S1: Linearity Analysis

**Analytes**		**Cancer Antigen 125 (CA-125)**	**Cancer Antigen 125 (CA-125)**		**Carcinoembryonic Antigen (CEA)**	**Carcinoembryonic Antigen (CEA)**		**CD5 (CD5L)**	**CD5 (CD5L)**		**Clusterin (CLU)**	**Clusterin (CLU)**		**Complement C3 (C3)**	**Complement C3 (C3)**		**Complement Factor H**	**Complement Factor H**	
**Units**			U/mL			ng/mL			ng/ml			ug/ml			mg/mL			ug/ml	
**Multiplex**			HMP1			HMP1			HMPC46			HMPC47			HMP3			HMPC46	
**LLOQ**			6.4			1.2			0.037			0.0029			1.6E-07			0.013	
**<u>Samples</u>**	**Linearity %**	**MFI**	**Result**	**Linearity %**	**MFI**	**Result**	**Linearity %**	**MFI**	**Result**	**Linearity %**	**MFI**	**Result**	**Linearity %**	**MFI**	**Result**	**Linearity %**	**MFI**	**Result**	**Linearity %**
**10X 10 {1:2}**		QNS	QNS		QNS	QNS		QNS	QNS		QNS	QNS		QNS	QNS		QNS	QNS	
**10X 10 {1:6}**		5.0	-		2.0	-		1794	5.7		645	1.0		2179	0.00019		4644	3.2	
**10X 10 {1:18}**	-	3.0	-	-	2.0	-	-	833	2.7	72%	252	0.38	88%	2407	0.00023	28%	1808	1.2	91%
**10X 10 {1:54}**	-	5.0	-	-	2.0	-	-	323	1.1	83%	97	0.14	88%	1434	0.00010	74%	551	0.40	95%
**10X 10 {1:162}**	-	2.0	-	-	3.0	-	-	116	0.40	89%	46	0.063	76%	679	3.7E-05	92%	192	0.16	85%
**10X 10 {1:486}**	-	2.0	-	-	3.0	-	-	52	0.17	77%	23	0.027	78%	221	9.8E-06	126%	69	0.046	115%
**10X 10 {1:1458}**	-	4.0	-	-	3.0	-	-	20	0.056	104%	14	0.012	71%	80	3.2E-06	103%	32	-	-
**10X 10 {1:4374}**	-	4.0	-	-	2.0	-	-	14	-	-	13	0.011	38%	33	1.1E-06	93%	18	-	-
**10X 10 {1:13,122}**	-	3.0	-	-	2.0	-	-	14	-	-	9.0	0.0051	72%	13	2.7E-07	144%	15	-	-
**10X 10 {1:39,366}**	-	2.0	-	-	2.0	-	-	14	-	-	8.0	0.0037	45%	10	-	-	13	-	-
**10X 10 {1:118,098}**	-	3.0	-	-	2.5	-	-	11	-	-	8.0	0.0037	33%	8.0	-	-	12	-	-
**10X 10 {1:354,294}**	-	2.5	-	-	3.0	-	-	11	-	-	9.0	0.0051	24%	7.0	-	-	11	-	-
**2X2 {1:2}**		QNS	QNS		QNS	QNS		QNS	QNS		QNS	QNS		QNS	QNS		QNS	QNS	
**2X2 {1:6}**		4.0	-		3.0	-		11143	56		1802	3.6		1778	0.00014		21050	-	
**2X2 {1:18}**	-	4.0	-	-	3.0	-	-	7700	32	58%	1051	1.8	68%	2841	0.00030	16%	15226	-	-
**2X2 {1:54}**	-	4.0	-	-	4.0	-	-	3535	12	89%	356	0.54	110%	2498	0.00024	42%	8280	7.2	-
**2X2 {1:162}**	-	4.0	-	-	3.0	-	-	1298	4.1	98%	106	0.16	114%	1384	9.7E-05	84%	3367	2.2	110%
**2X2 {1:486}**	-	3.0	-	-	5.0	-	-	398	1.3	105%	39	0.052	101%	584	3.1E-05	106%	971	0.66	110%
**2X2 {1:1458}**	-	5.0	-	-	4.0	-	-	152	0.52	83%	18	0.019	92%	199	8.7E-06	118%	295	0.23	94%
**2X2 {1:4374}**	-	3.0	-	-	2.0	-	-	53	0.18	97%	13	0.010	61%	75	3.0E-06	98%	105	0.083	95%
**2X2 {1:13,122}**	-	3.0	-	-	3.0	-	-	27	0.082	73%	10	0.0065	52%	27	8.9E-07	111%	42	-	-
**2X2 {1:39,366}**	-	3.0	-	-	3.0	-	-	15	0.037	74%	9.0	0.0051	43%	14	3.3E-07	89%	21	-	-
**2X2 {1:118,098}**	-	2.5	-	-	2.0	-	-	15	-	-	10	0.0065	26%	10	-	-	14	-	-
**2X2 {1:354,294}**	-	4.0	-	-	3.0	-	-	13	-	-	8.0	0.0037	58%	8.0	-	-	13	-	-

Samples were obtained from diseased sample pool. HDL was isolated by 10X10 or 2X2 centrifugation technique. MFI refers to Median Fluorescent Intensity, is based on beads with unique fluorescent signatures.The LLOQ (Lower Limit of Quantitation) is the lowest concentration of an analyte in a sample that can be reliably detected and at which the total error meets the laboratory's requirements for accuracy. In this case, the laboratory's requirement for accuracy is the concentration of an analyte at which the coefficient of variation of replicate standard samples is 30%. QNS indicates Quantity Not Sufficient for testing. Linearity % calculated as initial result divided by next dilution result multiplied by 3. Results below the LLOQ have been omitted and replaced with "-". <HIGH> results greater than the assay range have been omitted and replaced with "-".Results highlighted in blue reflect acceptable dilution linearity as defined as between 70-130% recovery. Analytes without highlighted blue results reflect either lack of detection in the samples or lack of linearity between dilutions.

Table S1: Linearity Analysis

Analytes	C-Reactive Protein (CRP)	C-Reactive Protein (CRP)		Creatine Kinase-MB (CK-MB)	Creatine Kinase-MB (CK-MB)		EN-RAGE	EN-RAGE		Factor VII	Factor VII		Fatty Acid-Binding Protein, heart (FABP, heart)	Fatty Acid-Binding Protein, heart (FABP, heart)		Ferritin (FRTN)	Ferritin (FRTN)		Fetuin-A
Units		ug/mL			ng/mL			ng/mL			ng/mL			ng/mL			ng/mL		
Multiplex		HMP2			HMP1			HMP10			HMP1			HMP1			HMP8		
LLOQ		6.5E-05			0.42			0.050			2.0			7.7			0.086		
Samples	MFI	Result	Linearity %	MFI	Result	Linearity %	MFI	Result	Linearity %	MFI	Result	Linearity %	MFI	Result	Linearity %	MFI	Result	Linearity %	MFI
10X 10 {1:2}	QNS	QNS		QNS	QNS		QNS	QNS		QNS	QNS		QNS	QNS		QNS	QNS		QNS
10X 10 {1:6}	4899	-		5.0	-		12	-		2.5	-		3.0	-		23	0.22		10875
10X 10 {1:18}	1894	0.0090	-	3.0	-	-	8.0	-	-	3.0	-	-	3.0	-	-	11	-	-	9585
10X 10 {1:54}	549	0.0025	120%	5.0	-	-	8.0	-	-	4.0	-	-	4.0	-	-	8.0	-	-	5931
10X 10 {1:162}	241	0.0011	79%	4.0	-	-	6.0	-	-	3.0	-	-	3.0	-	-	7.0	-	-	2234
10X 10 {1:486}	77	0.00023	152%	3.0	-	-	6.0	-	-	2.0	-	-	4.0	-	-	8.0	-	-	669
10X 10 {1:1458}	42	-	-	3.0	-	-	5.0	-	-	2.0	-	-	3.0	-	-	7.5	-	-	208
10X 10 {1:4374}	26	-	-	3.5	-	-	3.5	-	-	3.0	-	-	3.0	-	-	6.0	-	-	83
10X 10 {1:13,122}	29	-	-	3.0	-	-	4.0	-	-	2.0	-	-	4.0	-	-	10	-	-	42
10X 10 {1:39,366}	20	-	-	4.0	-	-	4.0	-	-	2.0	-	-	4.0	-	-	7.0	-	-	32
10X 10 {1:118,098}	27	-	-	3.0	-	-	2.0	-	-	1.0	-	-	3.5	-	-	8.0	-	-	34
10X 10 {1:354,294}	21	-	-	2.5	-	-	6.0	-	-	3.0	-	-	2.0	-	-	8.0	-	-	27
2X2 {1:2}	QNS	QNS		QNS	QNS		QNS	QNS		QNS	QNS		QNS	QNS		QNS	QNS		QNS
2X2 {1:6}	10692	-		5.0	-		31	0.14		24	9.4		8.5	-		37	0.44		10389
2X2 {1:18}	9037	-	-	3.0	-	-	20	0.070	64%	7.0	2.2	143%	5.0	-	-	11	-	-	10611
2X2 {1:54}	3505	0.018	-	5.0	-	-	25	0.10	23%	3.0	-	-	4.0	-	-	11	-	-	10059
2X2 {1:162}	1028	0.0048	124%	4.0	-	-	24	0.095	35%	4.0	-	-	3.0	-	-	5.5	-	-	7396
2X2 {1:486}	387	0.0018	91%	5.0	-	-	17	0.056	56%	2.0	-	-	3.0	-	-	6.5	-	-	3384
2X2 {1:1458}	115	0.00043	137%	5.0	-	-	7.0	-	-	4.0	-	-	5.0	-	-	8.0	-	-	1071
2X2 {1:4374}	65	0.00017	84%	3.0	-	-	7.0	-	-	3.0	-	-	5.0	-	-	9.0	-	-	320
2X2 {1:13,122}	38	-	-	4.0	-	-	3.0	-	-	4.0	-	-	3.0	-	-	6.5	-	-	121
2X2 {1:39,366}	32	-	-	4.0	-	-	9.0	-	-	2.0	-	-	3.0	-	-	7.0	-	-	57
2X2 {1:118,098}	28	-	-	4.0	-	-	3.0	-	-	2.0	-	-	4.0	-	-	8.0	-	-	31
2X2 {1:354,294}	29	-	-	4.0	-	-	4.0	-	-	3.0	-	-	4.0	-	-	6.5	-	-	33

Samples were obtained from diseased sample pool. HDL was isolated by 10X10 or 2X2 centrifugation technique. MFI refers to Median Fluorescent Intensity, is based on beads with unique fluorescent signatures.The LLOQ (Lower Limit of Quantitation) is the lowest concentration of an analyte in a sample that can be reliably detected and at which the total error meets the laboratory’s requirements for accuracy. In this case, the laboratory’s requirement for accuracy is the concentration of an analyte at which the coefficient of variation of replicate standard samples is 30%. QNS indicates Quantity Not Sufficient for testing. Linearity % calculated as initial result divided by next dilution result multiplied by 3. Results below the LLOQ have been omitted and replaced with "-". <HIGH> results greater than the assay range have been omitted and replaced with "-".Results highlighted in blue reflect acceptable dilution linearity as defined as between 70-130% recovery. Analytes without highlighted blue results reflect either lack of detection in the samples or lack of linearity between dilutions.

Table S1: Linearity Analysis

Analytes	Fetuin-A		Fibrinogen	Fibrinogen		Growth Hormone (GH)	Growth Hormone (GH)		Haptoglobin	Haptoglobin		Immunoglobulin A (IgA)	Immunoglobulin A (IgA)		Immunoglobulin E (IgE)	Immunoglobulin E (IgE)		Immunoglobulin M (IGM)	Immunoglobulin M (IGM)
**Units**	ug/ml			mg/mL			ng/mL			mg/mL			mg/mL			U/mL			mg/mL
**Multiplex**	HMPC46			HMP3			HMP1			HMP3			HMP3			HMP1			HMP3
**LLOQ**	0.0024			2.0E-06			0.26			3.9E-07			1.2E-06			26			1.7E-06
**Samples**	**Result**	**Linearity %**	**MFI**	**Result**	**Linearity %**	**MFI**	**Result**	**Linearity %**	**MFI**	**Result**	**Linearity %**	**MFI**	**Result**	**Linearity %**	**MFI**	**Result**	**Linearity %**	**MFI**	**Result**
**10X 10 {1:2}**	QNS		QNS	QNS		QNS	QNS		QNS	QNS		QNS	QNS		QNS	QNS		QNS	QNS
**10X 10 {1:6}**	-		998	0.00044		4.0	-		2428	0.00024		4465	0.00042		4.0	-		1621	0.00090
**10X 10 {1:18}**	-	-	783	0.00030	48%	2.0	-	-	1504	0.00014	59%	2391	0.00021	68%	6.0	-	-	712	0.00041
**10X 10 {1:54}**	-	-	307	0.00011	96%	2.0	-	-	607	4.9E-05	92%	663	5.7E-05	121%	6.5	-	-	212	0.00013
**10X 10 {1:162}**	0.60	-	123	4.4E-05	80%	2.0	-	-	218	1.6E-05	101%	232	2.0E-05	94%	5.5	-	-	70	4.4E-05
**10X 10 {1:486}**	0.18	111%	47	1.7E-05	84%	3.0	-	-	76	4.9E-06	111%	83	5.8E-06	116%	6.0	-	-	24	1.4E-05
**10X 10 {1:1458}**	0.062	97%	23	8.1E-06	71%	2.0	-	-	36	1.8E-06	91%	54	2.8E-06	70%	4.0	-	-	11	5.3E-06
**10X 10 {1:4374}**	0.024	86%	12	3.5E-06	77%	1.0	-	-	18	4.3E-07	138%	35	-	-	4.5	-	-	5.0	-
**10X 10 {1:13,122}**	0.010	78%	8.0	-	-	2.0	-	-	15	-	-	30	-	-	4.5	-	-	4.0	-
**10X 10 {1:39,366}**	0.0066	51%	5.0	-	-	1.0	-	-	12	-	-	28	-	-	4.0	-	-	2.0	-
**10X 10 {1:118,098}**	0.0075	30%	4.0	-	-	2.0	-	-	9.0	-	-	30	-	-	5.0	-	-	3.0	-
**10X 10 {1:354,294}**	0.0050	50%	4.5	-	-	2.0	-	-	9.0	-	-	29	-	-	4.0	-	-	2.0	-
**2X2 {1:2}**	QNS		QNS	QNS		QNS	QNS		QNS	QNS		QNS	QNS		QNS	QNS		QNS	QNS
**2X2 {1:6}**	-		1030	0.00046		3.0	-		1813	0.00017		2427	0.00021		5.0	-		5274	0.0029
**2X2 {1:18}**	-	-	885	0.00036	43%	3.0	-	-	3053	0.00032	18%	5997	0.00060	12%	7.0	-	-	2046	0.0011
**2X2 {1:54}**	-	-	472	0.00016	73%	1.0	-	-	2245	0.00022	49%	5311	0.00052	39%	6.0	-	-	605	0.00035
**2X2 {1:162}**	-	-	177	6.1E-05	89%	2.0	-	-	866	7.2E-05	101%	2162	0.00018	93%	4.5	-	-	165	0.00010
**2X2 {1:486}**	1.1	-	71	2.6E-05	79%	3.0	-	-	288	2.2E-05	110%	646	5.5E-05	111%	8.0	-	-	62	3.9E-05
**2X2 {1:1458}**	0.28	131%	33	1.2E-05	72%	2.0	-	-	113	7.9E-06	93%	203	1.7E-05	106%	8.0	-	-	24	1.4E-05
**2X2 {1:4374}**	0.093	100%	17	5.7E-06	71%	1.0	-	-	40	2.1E-06	125%	84	5.9E-06	98%	3.0	-	-	8.5	3.9E-06
**2X2 {1:13,122}**	0.036	86%	9.0	2.2E-06	88%	2.0	-	-	20	5.8E-07	120%	46	1.9E-06	104%	4.0	-	-	5.0	-
**2X2 {1:39,366}**	0.015	79%	6.0	-	-	1.0	-	-	12	-	-	38	-	-	3.0	-	-	4.0	-
**2X2 {1:118,098}**	0.0065	79%	5.0	-	-	3.0	-	-	13	-	-	29	-	-	5.0	-	-	3.0	-
**2X2 {1:354,294}**	0.0071	30%	6.0	-	-	3.0	-	-	12	-	-	26	-	-	6.0	-	-	3.0	-

Samples were obtained from diseased sample pool. HDL was isolated by 10X10 or 2X2 centrifugation technique. MFI refers to Median Fluorescent Intensity, is based on beads with unique fluorescent signatures.The LLOQ (Lower Limit of Quantitation) is the lowest concentration of an analyte in a sample that can be reliably detected and at which the total error meets the laboratory's requirements for accuracy. In this case, the laboratory's requirement for accuracy is the concentration of an analyte at which the coefficient of variation of replicate standard samples is 30%. QNS indicates Quantity Not Sufficient for testing. Linearity % calculated as initial result divided by next dilution result multiplied by 3. Results below the LLOQ have been omitted and replaced with "-". <HIGH> results greater than the assay range have been omitted and replaced with "-".Results highlighted in blue reflect acceptable dilution linearity as defined as between 70-130% recovery. Analytes without highlighted blue results reflect either lack of detection in the samples or lack of linearity between dilutions.

Table S1: Linearity Analysis

**Analytes**		**Intercellular Adhesion Molecule 1 (ICAM-1)**	**Intercellular Adhesion Molecule 1 (ICAM-1)**		**Interleukin-1 beta (IL-1 beta)**	**Interleukin-1 beta (IL-1 beta)**		**Interleukin-6 (IL-6)**	**Interleukin-6 (IL-6)**		**Leptin**	**Leptin**		**Matrix Metalloproteinase-3 (MMP-3)**	**Matrix Metalloproteinase-3 (MMP-3)**		**Matrix Metalloproteinase-9 (MMP-9)**	**Matrix Metalloproteinase-9 (MMP-9)**	
**Units**			ng/mL			pg/mL			pg/mL			ng/mL			ng/mL			ng/mL	
**Multiplex**			HMP1			HMP1			HMP1			HMP1			HMP1			HMP1	
**LLOQ**			5.2			1.9			4.7			0.11			0.28			42	
**Samples**	**Linearity %**	**MFI**	**Result**	**Linearity %**	**MFI**	**Result**	**Linearity %**	**MFI**	**Result**	**Linearity %**	**MFI**	**Result**	**Linearity %**	**MFI**	**Result**	**Linearity %**	**MFI**	**Result**	**Linearity %**
**10X 10 {1:2}**		QNS	QNS		QNS	QNS		QNS	QNS		QNS	QNS		QNS	QNS		QNS	QNS	
**10X 10 {1:6}**		4.0	-		4.0	-		4.0	-		155	1.7		17	-		3.0	-	
**10X 10 {1:18}**	73%	3.0	-	-	4.0	-	-	2.0	-	-	34	0.62	89%	13	-	-	3.0	-	-
**10X 10 {1:54}**	104%	5.0	-	-	2.0	-	-	1.0	-	-	7.5	0.18	115%	9.0	-	-	2.0	-	-
**10X 10 {1:162}**	98%	3.0	-	-	1.0	-	-	2.0	-	-	3.0	-	-	9.0	-	-	3.0	-	-
**10X 10 {1:486}**	102%	3.0	-	-	1.0	-	-	2.0	-	-	3.0	-	-	8.0	-	-	3.0	-	-
**10X 10 {1:1458}**	91%	2.5	-	-	3.0	-	-	2.0	-	-	2.0	-	-	9.0	-	-	2.0	-	-
**10X 10 {1:4374}**	-	2.0	-	-	1.0	-	-	1.0	-	-	3.0	-	-	10	-	-	2.0	-	-
**10X 10 {1:13,122}**	-	2.0	-	-	3.0	-	-	2.0	-	-	2.0	-	-	9.0	-	-	3.0	-	-
**10X 10 {1:39,366}**	-	4.0	-	-	2.0	-	-	3.0	-	-	2.0	-	-	8.0	-	-	2.0	-	-
**10X 10 {1:118,098}**	-	5.0	-	-	2.0	-	-	3.0	-	-	2.0	-	-	8.0	-	-	3.5	-	-
**10X 10 {1:354,294}**	-	4.0	-	-	3.0	-	-	2.0	-	-	3.0	-	-	9.0	-	-	3.0	-	-
**2X2 {1:2}**		QNS	QNS		QNS	QNS		QNS	QNS		QNS	QNS		QNS	QNS		QNS	QNS	
**2X2 {1:6}**		15	7.0		2.0	-		4.0	-		816	4.9		78	0.74		3.0	-	
**2X2 {1:18}**	85%	6.0	-	-	2.0	-	-	4.0	-	-	146	1.6	102%	31	-	-	2.0	-	-
**2X2 {1:54}**	107%	3.0	-	-	3.0	-	-	3.0	-	-	27	0.53	100%	19	-	-	3.0	-	-
**2X2 {1:162}**	115%	4.0	-	-	3.0	-	-	3.0	-	-	7.0	0.17	106%	11	-	-	4.0	-	-
**2X2 {1:486}**	88%	5.0	-	-	2.0	-	-	3.0	-	-	5.0	0.11	51%	11	-	-	5.0	-	-
**2X2 {1:1458}**	90%	7.0	-	-	3.0	-	-	2.5	-	-	4.0	-	-	9.5	-	-	3.0	-	-
**2X2 {1:4374}**	123%	3.0	-	-	2.0	-	-	3.0	-	-	2.0	-	-	11	-	-	3.0	-	-
**2X2 {1:13,122}**	-	2.0	-	-	2.0	-	-	2.0	-	-	2.0	-	-	7.0	-	-	2.0	-	-
**2X2 {1:39,366}**	-	3.0	-	-	1.0	-	-	2.0	-	-	2.0	-	-	10	-	-	2.0	-	-
**2X2 {1:118,098}**	-	3.0	-	-	2.0	-	-	1.0	-	-	3.0	-	-	8.0	-	-	3.0	-	-
**2X2 {1:354,294}**	-	3.0	-	-	2.0	-	-	2.0	-	-	1.0	-	-	9.0	-	-	2.5	-	-

Samples were obtained from diseased sample pool. HDL was isolated by 10X10 or 2X2 centrifugation technique. MFI refers to Median Fluorescent Intensity, is based on beads with unique fluorescent signatures.The LLOQ (Lower Limit of Quantitation) is the lowest concentration of an analyte in a sample that can be reliably detected and at which the total error meets the laboratory's requirements for accuracy. In this case, the laboratory's requirement for accuracy is the concentration of an analyte at which the coefficient of variation of replicate standard samples is 30%. QNS indicates Quantity Not Sufficient for testing. Linearity % calculated as initial result divided by next dilution result multiplied by 3. Results below the LLOQ have been omitted and replaced with "-". <HIGH> results greater than the assay range have been omitted and replaced with "-".Results highlighted in blue reflect acceptable dilution linearity as defined as between 70-130% recovery. Analytes without highlighted blue results reflect either lack of detection in the samples or lack of linearity between dilutions.

Table S1: Linearity Analysis

Analytes	Myeloperoxidase (MPO)	Myeloperoxidase (MPO)		Myoglobin	Myoglobin		Plasminogen Activator Inhibitor 1 (PAI-1)	Plasminogen Activator Inhibitor 1 (PAI-1)		Prostate-Specific Antigen, Free (PSA-f)	Prostate-Specific Antigen, Free (PSA-f)		Prostatic Acid Phosphatase (PAP)	Prostatic Acid Phosphatase (PAP)		Serum Amyloid P-Component (SAP)	Serum Amyloid P-Component (SAP)		Sex Hormone-Binding Globulin (SHBG)	Sex Hormone-Binding Globulin (SHBG)
**Units**		ng/mL			ng/mL			ng/mL			ng/mL			ng/mL			ug/mL			nmol/L
**Multiplex**		HMP10			HMP8			HMP8			HMP1			HMP1			HMP2			HMP2
**LLOQ**		18			0.20			0.19			0.024			0.21			5.6E-05			0.0071
**Samples**	**MFI**	**Result**	**Linearity %**	**MFI**	**Result**	**Linearity %**	**MFI**	**Result**	**Linearity %**	**MFI**	**Result**	**Linearity %**	**MFI**	**Result**	**Linearity %**	**MFI**	**Result**	**Linearity %**	**MFI**	**Result**
**10X 10 {1:2}**	QNS	QNS		QNS	QNS		QNS	QNS		QNS	QNS		QNS	QNS		QNS	QNS		QNS	QNS
**10X 10 {1:6}**	8.0	19		3533	15		176	0.51		11	-		10	-		4153	0.045		15	0.014
**10X 10 {1:18}**	4.0	-	-	901	5.2	97%	100	-	-	14	-	-	10	-	-	1810	0.018	81%	10	0.0078
**10X 10 {1:54}**	1.5	-	-	189	1.6	111%	64	-	-	12	-	-	10	-	-	612	0.0060	102%	6.0	-
**10X 10 {1:162}**	2.0	-	-	64	0.51	101%	62	-	-	9.5	-	-	9.0	-	-	216	0.0019	103%	6.0	-
**10X 10 {1:486}**	2.5	-	-	35	-	-	59	-	-	9.0	-	-	9.5	-	-	63	0.00045	144%	3.0	-
**10X 10 {1:1458}**	2.0	-	-	26	-	-	53	-	-	7.0	-	-	11	-	-	22	0.00011	138%	2.5	-
**10X 10 {1:4374}**	1.0	-	-	19	-	-	51	-	-	9.0	-	-	10	-	-	7.5	-	-	1.0	-
**10X 10 {1:13,122}**	2.5	-	-	19	-	-	55	-	-	11	-	-	10	-	-	4.0	-	-	4.0	-
**10X 10 {1:39,366}**	2.5	-	-	20	-	-	49	-	-	10	-	-	10	-	-	3.0	-	-	2.0	-
**10X 10 {1:118,098}**	3.0	-	-	16	-	-	47	-	-	10	-	-	11	-	-	4.0	-	-	3.0	-
**10X 10 {1:354,294}**	2.5	-	-	18	-	-	56	-	-	9.5	-	-	9.5	-	-	2.0	-	-	1.0	-
**2X2 {1:2}**	QNS	QNS		QNS	QNS		QNS	QNS		QNS	QNS		QNS	QNS		QNS	QNS		QNS	QNS
**2X2 {1:6}**	6.0	-		6784	28		973	4.0		10	-		10	-		7344	0.12		700	0.51
**2X2 {1:18}**	4.0	-	-	2093	9.8	95%	373	1.4	95%	9.5	-	-	9.5	-	-	4516	0.049	78%	171	0.17
**2X2 {1:54}**	3.0	-	-	525	3.5	94%	179	0.52	89%	10	-	-	12	-	-	2035	0.021	79%	71	0.076
**2X2 {1:162}**	4.0	-	-	111	0.96	121%	96	-	-	8.5	-	-	9.5	-	-	651	0.0064	108%	23	0.024
**2X2 {1:486}**	4.0	-	-	48	0.35	91%	64	-	-	11	-	-	11	-	-	201	0.0018	119%	7.0	-
**2X2 {1:1458}**	3.0	-	-	24	-	-	53	-	-	8.0	-	-	12	-	-	76	0.00057	105%	6.0	-
**2X2 {1:4374}**	2.0	-	-	20	-	-	62	-	-	9.0	-	-	11	-	-	23	0.00012	164%	2.0	-
**2X2 {1:13,122}**	3.5	-	-	21	-	-	54	-	-	8.0	-	-	9.0	-	-	8.0	-	-	2.0	-
**2X2 {1:39,366}**	5.0	-	-	19	-	-	54	-	-	9.0	-	-	10	-	-	4.0	-	-	2.0	-
**2X2 {1:118,098}**	3.0	-	-	18	-	-	53	-	-	8.0	-	-	10	-	-	2.5	-	-	3.0	-
**2X2 {1:354,294}**	2.0	-	-	17	-	-	49	-	-	9.5	-	-	9.0	-	-	2.0	-	-	2.0	-

Samples were obtained from diseased sample pool. HDL was isolated by 10X10 or 2X2 centrifugation technique. MFI refers to Median Fluorescent Intensity, is based on beads with unique fluorescent signatures.The LLOQ (Lower Limit of Quantitation) is the lowest concentration of an analyte in a sample that can be reliably detected and at which the total error meets the laboratory's requirements for accuracy. In this case, the laboratory's requirement for accuracy is the concentration of an analyte at which the coefficient of variation of replicate standard samples is 30%. QNS indicates Quantity Not Sufficient for testing. Linearity % calculated as initial result divided by next dilution result multiplied by 3. Results below the LLOQ have been omitted and replaced with "-". <HIGH> results greater than the assay range have been omitted and replaced with "-".Results highlighted in blue reflect acceptable dilution linearity as defined as between 70-130% recovery. Analytes without highlighted blue results reflect either lack of detection in the samples or lack of linearity between dilutions.

Table S1: Linearity Analysis

Analytes		T-Cell-Specific Protein RANTES (RANTES)	T-Cell-Specific Protein RANTES (RANTES)		Thrombospondin-1	Thrombospondin-1		Thyroid-Stimulating Hormone (TSH)	Thyroid-Stimulating Hormone (TSH)		Thyroxine-Binding Globulin (TBG)	Thyroxine-Binding Globulin (TBG)		Tissue Factor (TF)	Tissue Factor (TF)		Tissue Inhibitor of Metalloproteinases 1 (TIMP-1)	Tissue Inhibitor of Metalloproteinases 1 (TIMP-1)		Tumor Necrosis Factor alpha (TNF-alpha)	Tumor Necrosis Factor alpha (TNF-alpha)
Units			ng/mL			ng/mL			uIU/mL			ug/mL			ng/mL			ng/mL			pg/mL
Multiplex			HMP8			HMP10			HMP1			HMP2			HMP1			HMP8			HMP1
LLOQ			0.0019			2.5			0.036			0.00013			0.22			0.068			68
**Samples**	**Linearity %**	**MFI**	**Result**	**Linearity %**	**MFI**	**Result**	**Linearity %**	**MFI**	**Result**	**Linearity %**	**MFI**	**Result**	**Linearity %**	**MFI**	**Result**	**Linearity %**	**MFI**	**Result**	**Linearity %**	**MFI**	**Result**
**10X 10 {1:2}**		QNS	QNS		QNS	QNS		QNS	QNS		QNS	QNS		QNS	QNS		QNS	QNS		QNS	QNS
**10X 10 {1:6}**		2715	0.38		24	3.6		3.0	-		8420	0.32		4.0	-		1093	1.1		1.0	-
**10X 10 {1:18}**	59%	913	0.13	97%	11	-	-	2.0	-	-	2314	0.11	98%	3.0	-	-	248	0.39	93%	2.0	-
**10X 10 {1:54}**	-	247	0.035	125%	9.0	-	-	1.0	-	-	526	0.036	99%	3.0	-	-	58	0.12	108%	3.0	-
**10X 10 {1:162}**	-	96	0.012	100%	9.0	-	-	2.0	-	-	154	0.013	93%	3.0	-	-	31	-	-	2.0	-
**10X 10 {1:486}**	-	44	0.0035	111%	8.0	-	-	3.0	-	-	43	0.0033	133%	3.0	-	-	23	-	-	1.0	-
**10X 10 {1:1458}**	-	25	-	-	8.0	-	-	2.0	-	-	18	0.00074	147%	2.0	-	-	23	-	-	3.0	-
**10X 10 {1:4374}**	-	22	-	-	6.5	-	-	2.0	-	-	8.0	-	-	3.0	-	-	19	-	-	2.0	-
**10X 10 {1:13,122}**	-	20	-	-	9.0	-	-	1.0	-	-	10	-	-	4.0	-	-	22	-	-	2.0	-
**10X 10 {1:39,366}**	-	22	-	-	5.5	-	-	1.0	-	-	4.0	-	-	3.0	-	-	20	-	-	2.0	-
**10X 10 {1:118,098}**	-	18	-	-	9.0	-	-	3.0	-	-	8.5	-	-	2.0	-	-	19	-	-	1.5	-
**10X 10 {1:354,294}**	-	18	-	-	8.0	-	-	3.0	-	-	5.0	-	-	3.0	-	-	20	-	-	2.0	-
**2X2 {1:2}**		QNS	QNS		QNS	QNS		QNS	QNS		QNS	QNS		QNS	QNS		QNS	QNS		QNS	QNS
**2X2 {1:6}**		9610	1.5		21	8.0		17	0.12		16387	-		2.0	-		13811	14		2.0	-
**2X2 {1:18}**	103%	3494	0.49	104%	14	2.8	97%	7.0	-	-	14947	-	-	3.0	-	-	4299	3.3	139%	3.0	-
**2X2 {1:54}**	73%	1172	0.17	99%	12	-	-	4.0	-	-	7670	0.29	-	2.5	-	-	1176	1.1	97%	2.0	-
**2X2 {1:162}**	108%	336	0.048	115%	10	-	-	3.0	-	-	1927	0.094	103%	4.0	-	-	222	0.36	106%	2.0	-
**2X2 {1:486}**	-	116	0.015	109%	9.0	-	-	2.0	-	-	424	0.031	103%	3.5	-	-	63	0.13	93%	2.0	-
**2X2 {1:1458}**	-	48	0.0041	120%	12	-	-	4.0	-	-	119	0.010	100%	4.0	-	-	30	-	-	3.0	-
**2X2 {1:4374}**	-	31	-	-	10	-	-	2.0	-	-	34	0.0023	144%	3.5	-	-	28	-	-	2.0	-
**2X2 {1:13,122}**	-	23	-	-	9.0	-	-	1.0	-	-	14	0.00036	216%	3.0	-	-	22	-	-	3.0	-
**2X2 {1:39,366}**	-	22	-	-	8.0	-	-	1.0	-	-	7.0	-	-	3.0	-	-	20	-	-	3.0	-
**2X2 {1:118,098}**	-	18	-	-	9.0	-	-	3.0	-	-	6.0	-	-	3.0	-	-	19	-	-	3.0	-
**2X2 {1:354,294}**	-	19	-	-	8.0	-	-	3.0	-	-	5.0	-	-	3.0	-	-	21	-	-	2.0	-

Samples were obtained from diseased sample pool. HDL was isolated by 10X10 or 2X2 centrifugation technique. MFI refers to Median Fluorescent Intensity, is based on beads with unique fluorescent signatures.The LLOQ (Lower Limit of Quantitation) is the lowest concentration of an analyte in a sample that can be reliably detected and at which the total error meets the laboratory's requirements for accuracy. In this case, the laboratory's requirement for accuracy is the concentration of an analyte at which the coefficient of variation of replicate standard samples is 30%. QNS indicates Quantity Not Sufficient for testing. Linearity % calculated as initial result divided by next dilution result multiplied by 3. Results below the LLOQ have been omitted and replaced with "-". <HIGH> results greater than the assay range have been omitted and replaced with "-".Results highlighted in blue reflect acceptable dilution linearity as defined as between 70-130% recovery. Analytes without highlighted blue results reflect either lack of detection in the samples or lack of linearity between dilutions.

Table S1: Linearity Analysis

Analytes		Tumor Necrosis Factor Receptor 2 (TNFR2)	Tumor Necrosis Factor Receptor 2 (TNFR2)		Vascular Cell Adhesion Molecule-1 (VCAM-1)	Vascular Cell Adhesion Molecule-1 (VCAM-1)		Vitamin K-Dependent Protein S (VKDPS)	Vitamin K-Dependent Protein S (VKDPS)		Vitronectin	Vitronectin		von Willebrand Factor (vWF)	von Willebrand Factor (vWF)	
**Units**			ng/mL			ng/mL			ug/ml			ug/ml			ug/mL	
**Multiplex**			HMP8			HMP8			HMPC46			HMPC46			HMP2	
**LLOQ**			0.014			0.15			8.5E-05			0.046			0.0059	
**<u>Samples</u>**	**Linearity %**	**MFI**	**Result**	**Linearity %**	**MFI**	**Result**	**Linearity %**	**MFI**	**Result**	**Linearity %**	**MFI**	**Result**	**Linearity %**	**MFI**	**Result**	**Linearity %**
**10X 10 {1:2}**		QNS	QNS		QNS	QNS		QNS	QNS		QNS	QNS		QNS	QNS	
**10X 10 {1:6}**		47	0.077		1040	9.2		912	0.018		188	1.8		91	0.065	
**10X 10 {1:18}**	-	21	0.025	104%	407	3.7	83%	548	0.012	52%	89	1.1	52%	46	0.028	79%
**10X 10 {1:54}**	-	11	-	-	136	1.1	112%	246	0.0059	66%	34	0.58	65%	25	0.011	83%
**10X 10 {1:162}**	-	9.0	-	-	78	0.46	78%	103	0.0027	72%	21	0.39	50%	19	0.0066	56%
**10X 10 {1:486}**	-	11	-	-	48	-	-	64	0.0018	52%	15	0.28	45%	9.0	-	-
**10X 10 {1:1458}**	-	10	-	-	45	-	-	35	0.00097	60%	14	0.26	36%	8.0	-	-
**10X 10 {1:4374}**	-	9.0	-	-	38	-	-	16	0.00038	86%	9.0	0.15	59%	6.0	-	-
**10X 10 {1:13,122}**	-	6.0	-	-	34	-	-	10	0.00020	63%	10	0.17	29%	7.0	-	-
**10X 10 {1:39,366}**	-	8.0	-	-	34	-	-	8.5	0.00015	44%	11	0.20	29%	7.0	-	-
**10X 10 {1:118,098}**	-	7.0	-	-	32	-	-	10	0.00020	25%	11	0.20	33%	5.0	-	-
**10X 10 {1:354,294}**	-	9.0	-	-	39	-	-	8.5	0.00015	44%	10	0.17	38%	7.0	-	-
**2X2 {1:2}**		QNS	QNS		QNS	QNS		QNS	QNS		QNS	QNS		QNS	QNS	
**2X2 {1:6}**		829	1.8		3095	28		5503	0.10		2789	9.1		97	0.071	
**2X2 {1:18}**	-	260	0.56	107%	1266	11	84%	2827	0.051	65%	676	3.8	81%	44	0.026	93%
**2X2 {1:54}**	-	90	0.17	108%	481	4.3	86%	1151	0.022	77%	127	1.4	90%	30	0.015	58%
**2X2 {1:162}**	-	31	0.044	131%	160	1.3	108%	360	0.0081	91%	41	0.67	70%	19	0.0069	72%
**2X2 {1:486}**	-	14	-	-	81	0.50	89%	130	0.0034	81%	17	0.32	69%	10	-	-
**2X2 {1:1458}**	-	10	-	-	45	-	-	50	0.0014	81%	13	0.24	44%	11	-	-
**2X2 {1:4374}**	-	8.0	-	-	43	-	-	16	0.00039	117%	11	0.19	43%	7.0	-	-
**2X2 {1:13,122}**	-	9.0	-	-	39	-	-	13	0.00028	47%	11	0.20	31%	6.0	-	-
**2X2 {1:39,366}**	-	8.0	-	-	33	-	-	6.0	-	-	9.0	0.15	45%	4.0	-	-
**2X2 {1:118,098}**	-	9.0	-	-	30	-	-	8.0	0.00013	-	9.0	0.15	33%	8.0	-	-
**2X2 {1:354,294}**	-	9.0	-	-	34	-	-	8.0	0.00013	33%	11	0.20	25%	5.0	-	-

Samples were obtained from diseased sample pool. HDL was isolated by 10X10 or 2X2 centrifugation technique. MFI refers to Median Fluorescent Intensity, is based on beads with unique fluorescent signatures.The LLOQ (Lower Limit of Quantitation) is the lowest concentration of an analyte in a sample that can be reliably detected and at which the total error meets the laboratory's requirements for accuracy. In this case, the laboratory's requirement for accuracy is the concentration of an analyte at which the coefficient of variation of replicate standard samples is 30%. QNS indicates Quantity Not Sufficient for testing. Linearity % calculated as initial result divided by next dilution result multiplied by 3. Results below the LLOQ have been omitted and replaced with "-". <HIGH> results greater than the assay range have been omitted and replaced with "-".Results highlighted in blue reflect acceptable dilution linearity as defined as between 70-130% recovery. Analytes without highlighted blue results reflect either lack of detection in the samples or lack of linearity between dilutions.

Table S2: MRM transitions used to screen for HDL proteins

Q1	Q3	Uniprot ID	Dwell	ID	DP	CE	Method
499.286	771.4	P14174	10	MacrophageMigrationInhibFactor.LLCGLLAER.S-120215-00008.2.y7.heavy	85	25	1
499.286	342.71		10	MacrophageMigrationInhibFactor.LLCGLLAER.S-120215-00008.2.b7++.heavy	85	24	1
499.286	498.28		10	MacrophageMigrationInhibFactor.LLCGLLAER.S-120215-00008.2.y4.heavy	85	23	1
499.286	385.2		10	MacrophageMigrationInhibFactor.LLCGLLAER.S-120215-00008.2.y3.heavy	85	21	1
499.286	387.21		10	MacrophageMigrationInhibFactor.LLCGLLAER.S-120215-00008.2.b4.heavy	85	22	1
494.286	761.4		10	MacrophageMigrationInhibFactor.LLCGLLAER.S-120215-00008.2.y7.light	85	25	1
494.286	342.71		10	MacrophageMigrationInhibFactor.LLCGLLAER.S-120215-00008.2.b7++.light	85	24	1
494.286	488.28		10	MacrophageMigrationInhibFactor.LLCGLLAER.S-120215-00008.2.y4.light	85	23	1
494.286	375.2		10	MacrophageMigrationInhibFactor.LLCGLLAER.S-120215-00008.2.y3.light	85	21	1
494.286	387.21		10	MacrophageMigrationInhibFactor.LLCGLLAER.S-120215-00008.2.b4.light	85	22	1
771.948	353.21	P11597	10	CholesterolEsterTransProtein.LFLSLLDFQITPK.S-120215-00004.2.y3.heavy	75	27	1
771.948	969.53		10	CholesterolEsterTransProtein.LFLSLLDFQITPK.S-120215-00004.2.y8.heavy	75	34	1
771.948	856.45		10	CholesterolEsterTransProtein.LFLSLLDFQITPK.S-120215-00004.2.y7.heavy	75	33	1
771.948	1169.65		10	CholesterolEsterTransProtein.LFLSLLDFQITPK.S-120215-00004.2.y10.heavy	75	36	1
771.948	741.42		10	CholesterolEsterTransProtein.LFLSLLDFQITPK.S-120215-00004.2.y6.heavy	75	36	1
767.948	345.21		10	CholesterolEsterTransProtein.LFLSLLDFQITPK.S-120215-00004.2.y3.light	75	27	1
767.948	961.53		10	CholesterolEsterTransProtein.LFLSLLDFQITPK.S-120215-00004.2.y8.light	75	34	1
767.948	848.45		10	CholesterolEsterTransProtein.LFLSLLDFQITPK.S-120215-00004.2.y7.light	75	33	1
767.948	1161.65		10	CholesterolEsterTransProtein.LFLSLLDFQITPK.S-120215-00004.2.y10.light	75	36	1
767.948	733.42		10	CholesterolEsterTransProtein.LFLSLLDFQITPK.S-120215-00004.2.y6.light	75	36	1
643.326	625.32	P08493	10	HumanMatrixGla.NANTFISPQQR.S-120215-00006.2.y5.heavy	80	27	1
643.326	738.4		10	HumanMatrixGla.NANTFISPQQR.S-120215-00006.2.y6.heavy	80	29	1
643.326	538.29		10	HumanMatrixGla.NANTFISPQQR.S-120215-00006.2.y4.heavy	80	30	1
643.326	401.18		10	HumanMatrixGla.NANTFISPQQR.S-120215-00006.2.b4.heavy	80	28	1
643.326	885.47		10	HumanMatrixGla.NANTFISPQQR.S-120215-00006.2.y7.heavy	80	31	1
638.326	615.32		10	HumanMatrixGla.NANTFISPQQR.S-120215-00006.2.y5.light	80	27	1
638.326	728.4		10	HumanMatrixGla.NANTFISPQQR.S-120215-00006.2.y6.light	80	29	1
638.326	528.29		10	HumanMatrixGla.NANTFISPQQR.S-120215-00006.2.y4.light	80	30	1
638.326	401.18		10	HumanMatrixGla.NANTFISPQQR.S-120215-00006.2.b4.light	80	28	1
638.326	875.47		10	HumanMatrixGla.NANTFISPQQR.S-120215-00006.2.y7.light	80	31	1
723.373	868.47	P11597	10	CholesterolEsterTransProtein.NVSEDLPLPTFSPTLLGDSR.S-101021-00018.3.y8.heavy	80	23	1
723.373	650.84		10	CholesterolEsterTransProtein.NVSEDLPLPTFSPTLLGDSR.S-101021-00018.3.y12++.heavy	80	22	1
723.373	444.2		10	CholesterolEsterTransProtein.NVSEDLPLPTFSPTLLGDSR.S-101021-00018.3.y4.heavy	80	23	1
723.373	434.7		10	CholesterolEsterTransProtein.NVSEDLPLPTFSPTLLGDSR.S-101021-00018.3.y8++.heavy	80	27	1
720.04	858.47		10	CholesterolEsterTransProtein.NVSEDLPLPTFSPTLLGDSR.S-101021-00018.3.y8.light	80	23	1
720.04	645.84		10	CholesterolEsterTransProtein.NVSEDLPLPTFSPTLLGDSR.S-101021-00018.3.y12++.light	80	22	1
720.04	434.2		10	CholesterolEsterTransProtein.NVSEDLPLPTFSPTLLGDSR.S-101021-00018.3.y4.light	80	23	1
720.04	429.74		10	CholesterolEsterTransProtein.NVSEDLPLPTFSPTLLGDSR.S-101021-00018.3.y8++.light	80	27	1
498.572	470.26	P55058	10	HumanPhospholipidTrans.EGHFYYNISEVK.S-120215-00001.3.y4.heavy	80	22	1

498.572	583.34		10	HumanPhospholipidTrans.EGHFYYNISEVK.S-120215-00001.3.y5.heavy	80	22	1
498.572	697.38		10	HumanPhospholipidTrans.EGHFYYNISEVK.S-120215-00001.3.y6.heavy	80	23	1
498.572	634.26		10	HumanPhospholipidTrans.EGHFYYNISEVK.S-120215-00001.3.b5.heavy	80	26	1
498.572	383.22		10	HumanPhospholipidTrans.EGHFYYNISEVK.S-120215-00001.3.y3.heavy	80	23	1
495.905	462.26		10	HumanPhospholipidTrans.EGHFYYNISEVK.S-120215-00001.3.y4.light	80	22	1
495.905	575.34		10	HumanPhospholipidTrans.EGHFYYNISEVK.S-120215-00001.3.y5.light	80	22	1
495.905	689.38		10	HumanPhospholipidTrans.EGHFYYNISEVK.S-120215-00001.3.y6.light	80	23	1
495.905	634.26		10	HumanPhospholipidTrans.EGHFYYNISEVK.S-120215-00001.3.b5.light	80	26	1
495.905	375.22		10	HumanPhospholipidTrans.EGHFYYNISEVK.S-120215-00001.3.y3.light	80	23	1
848.975	738.43	P11597	10	CholesterolEsterTransProtein.GVSLFDIINPEIITR.S-120215-00005.2.y6.heavy	90	42	1
848.975	852.47		10	CholesterolEsterTransProtein.GVSLFDIINPEIITR.S-120215-00005.2.y7.heavy	90	36	1
848.975	965.56		10	CholesterolEsterTransProtein.GVSLFDIINPEIITR.S-120215-00005.2.y8.heavy	90	40	1
848.975	732.39		10	CholesterolEsterTransProtein.GVSLFDIINPEIITR.S-120215-00005.2.b7.heavy	90	33	1
848.975	1193.67		10	CholesterolEsterTransProtein.GVSLFDIINPEIITR.S-120215-00005.2.y10.heavy	90	41	1
843.975	728.43		10	CholesterolEsterTransProtein.GVSLFDIINPEIITR.S-120215-00005.2.y6.light	90	42	1
843.975	842.47		10	CholesterolEsterTransProtein.GVSLFDIINPEIITR.S-120215-00005.2.y7.light	90	36	1
843.975	955.56		10	CholesterolEsterTransProtein.GVSLFDIINPEIITR.S-120215-00005.2.y8.light	90	40	1
843.975	732.39		10	CholesterolEsterTransProtein.GVSLFDIINPEIITR.S-120215-00005.2.b7.light	90	33	1
843.975	1183.67		10	CholesterolEsterTransProtein.GVSLFDIINPEIITR.S-120215-00005.2.y10.light	90	41	1
481.253	625.32	P08493	10	HumanMatrixGla.RNANTFISPQQR.S-120215-00007.3.y5.heavy	53	21	1
481.253	538.29		10	HumanMatrixGla.RNANTFISPQQR.S-120215-00007.3.y4.heavy	53	21	1
481.253	557.28		10	HumanMatrixGla.RNANTFISPQQR.S-120215-00007.3.b5.heavy	53	23	1
481.253	704.35		10	HumanMatrixGla.RNANTFISPQQR.S-120215-00007.3.b6.heavy	53	21	1
481.253	738.4		10	HumanMatrixGla.RNANTFISPQQR.S-120215-00007.3.y6.heavy	53	21	1
477.92	615.32		10	HumanMatrixGla.RNANTFISPQQR.S-120215-00007.3.y5.light	53	21	1
477.92	528.29		10	HumanMatrixGla.RNANTFISPQQR.S-120215-00007.3.y4.light	53	21	1
477.92	557.28		10	HumanMatrixGla.RNANTFISPQQR.S-120215-00007.3.b5.light	53	23	1
477.92	704.35		10	HumanMatrixGla.RNANTFISPQQR.S-120215-00007.3.b6.light	53	21	1
477.92	728.4		10	HumanMatrixGla.RNANTFISPQQR.S-120215-00007.3.y6.light	53	21	1
669.328	519.75	P55058	10	HumanPhospholipidTrans.AVEPQLQEEER.BET6086A01.2.y8++.heavy	75	29	1
669.328	1038.5		10	HumanPhospholipidTrans.AVEPQLQEEER.BET6086A01.2.y8.heavy	75	31	1
669.328	700.3		10	HumanPhospholipidTrans.AVEPQLQEEER.BET6086A01.2.y5.heavy	75	36	1
669.328	572.25		10	HumanPhospholipidTrans.AVEPQLQEEER.BET6086A01.2.y4.heavy	75	36	1
669.328	813.39		10	HumanPhospholipidTrans.AVEPQLQEEER.BET6086A01.2.y6.heavy	75	41	1
664.328	514.75		10	HumanPhospholipidTrans.AVEPQLQEEER.BET6086A01.2.y8++.light	75	29	1
664.328	1028.5		10	HumanPhospholipidTrans.AVEPQLQEEER.BET6086A01.2.y8.light	75	31	1
664.328	690.3		10	HumanPhospholipidTrans.AVEPQLQEEER.BET6086A01.2.y5.light	75	36	1
664.328	562.25		10	HumanPhospholipidTrans.AVEPQLQEEER.BET6086A01.2.y4.light	75	36	1
664.328	803.39		10	HumanPhospholipidTrans.AVEPQLQEEER.BET6086A01.2.y6.light	75	41	1
697.9	951.52	P04180	10	LCAT.S-110729-00003.SSGLVSNAPGVQIR.2.y9.heavy	66	36	1
692.9	941.52		10	LCAT.S-110729-00003.SSGLVSNAPGVQIR.2.y9.light	66	36	1

697.9	750.44		10	LCAT.S-110729-00003.SSGLVSNAPGVQIR.2.y7.heavy	66	37	1
692.9	740.44		10	LCAT.S-110729-00003.SSGLVSNAPGVQIR.2.y7.light	66	37	1
441.3	669.35		10	Apolipoprotein D.VLNQELR.S-090813-00010.2.y5.heavy	66	22	1
441.3	391.72	P05090	10	Apolipoprotein D.VLNQELR.S-090813-00010.2.y++6.heavy	66	21	1
436.25	659.35		10	Apolipoprotein D.VLNQELR.S-090813-00010.2.y5.light	66	22	1
436.25	386.72		10	Apolipoprotein D.VLNQELR.S-090813-00010.2.y++6.light	66	21	1
408.6	576.8	P02647	10	Apolipoprotein A-I.ATEHLSTLSEK.S-120309-00034.3.y10++.heavy	52	19	2
408.6	526.3		10	Apolipoprotein A-I.ATEHLSTLSEK.S-120309-00034.3.y9++.heavy	52	18	2
405.9	572.8		10	Apolipoprotein A-I.ATEHLSTLSEK.S-120309-00034.3.y10++.light	52	19	2
405.9	522.3		10	Apolipoprotein A-I.ATEHLSTLSEK.S-120309-00034.3.y9++.light	52	18	2
490.8	447.2	P02652	10	Apolipoprotein A-II precursor.SPELQAEAK.S-120309-00029.2.y++8.heavy	64	26	2
490.8	157.6		10	Apolipoprotein A-II precursor.SPELQAEAK.S-120309-00029.2.b++3.heavy	64	34	2
486.8	443.2		10	Apolipoprotein A-II precursor.SPELQAEAK.S-120309-00029.2.y++8.light	64	26	2
486.8	157.6		10	Apolipoprotein A-II precursor.SPELQAEAK.S-120309-00029.2.b++3.light	64	34	2
678.8	544.3	P06727	10	Apolipoprotein A-IV.SLAPYAQDTQEK.S-12030900024.2.y++9.heavy	67	30	2
678.8	1087.5		10	Apolipoprotein A-IV.SLAPYAQDTQEK.S-12030900024.2.y9.heavy	67	30	2
675.8	540.3		10	Apolipoprotein A-IV.SLAPYAQDTQEK.S-12030900024.2.y++9.light	67	30	2
675.8	1079.5		10	Apolipoprotein A-IV.SLAPYAQDTQEK.S-12030900024.2.y9.light	67	30	2
528.3	454.8	P04114	10	Apolipoprotein B-100.FPEVDVLTK.S-110708-00011.2.y++8.heavy	65	28	2
528.3	811.5		10	Apolipoprotein B-100.FPEVDVLTK.S-110708-00011.2.y7.heavy	65	26	2
524.3	450.8		10	Apolipoprotein B-100.FPEVDVLTK.S-110708-00011.2.y++8.light	65	28	2
524.3	803.5		10	Apolipoprotein B-100.FPEVDVLTK.S-110708-00011.2.y7.light	65	26	2
528.3	908.5		10	Apolipoprotein B-100.FPEVDVLTK.S-110708-00011.2.y8.heavy	65	27	2
524.3	900.5		10	Apolipoprotein B-100.FPEVDVLTK.S-110708-00011.2.y8.light	65	27	2
520.8	470.2	P02654	10	Apolipoprotein C-I lipoprotein.TPDVSSALDK.S-120309-00015.2.y++9.heavy	78	29	2
520.8	628.3		10	Apolipoprotein C-I lipoprotein.TPDVSSALDK.S-120309-00015.2.y6.heavy	78	29	2
516.8	466.2		10	Apolipoprotein C-I lipoprotein.TPDVSSALDK.S-120309-00015.2.y++9.light	78	29	2
516.8	620.3		10	Apolipoprotein C-I lipoprotein.TPDVSSALDK.S-120309-00015.2.y6.light	78	29	2
602.8	961.5	P02656	10	Apolipoprotein C-III.GWVTDGFSSLK.S-120309-00013.2.y9.heavy	59	25	2
602.8	244.1		10	Apolipoprotein C-III.GWVTDGFSSLK.S-120309-00013.2.b2.heavy	59	31	2
598.8	953.5		10	Apolipoprotein C-III.GWVTDGFSSLK.S-120309-00013.2.y9.light	59	25	2
598.8	244.1		10	Apolipoprotein C-III.GWVTDGFSSLK.S-120309-00013.2.b2.light	59	31	2
602.8	862.4		10	Apolipoprotein C-III.GWVTDGFSSLK.S-120309-00013.2.y8.heavy	59	26	2
598.8	854.4		10	Apolipoprotein C-III.GWVTDGFSSLK.S-120309-00013.2.y8.light	59	26	2
489.8	598.3	P02649	10	Apolipoprotein E.LGPLVEQGR.S-120309-00022.2.y5.heavy	70	30	2
489.8	499.2		10	Apolipoprotein E.LGPLVEQGR.S-120309-00022.2.y4.heavy	70	28	2
484.8	588.3		10	Apolipoprotein E.LGPLVEQGR.S-120309-00022.2.y5.light	70	30	2
484.8	489.2		10	Apolipoprotein E.LGPLVEQGR.S-120309-00022.2.y4.light	70	28	2
820.9	656.3	O14791	10	Apolipoprotein L1.VTEPISAESGEQVER.S-110124-00044.2.y++12.heavy	90	38	1
820.9	1101.5		10	Apolipoprotein L1.VTEPISAESGEQVER.S-110124-00044.2.y10.heavy	90	50	1
815.9	651.3		10	Apolipoprotein L1.VTEPISAESGEQVER.S-110124-00044.2.y++12.light	90	38	1

815.9	1091.5		10	Apolipoprotein L1.VTEPISAESGEQVER.S-110124-00044.2.y10.light	90	50	1
516.8	662.3	P02749	10	Beta-2-glycoprotein I_Apo H.ATVVYQGER.S-120309-00025.2.y5.heavy	65	25	2
516.8	761.4		10	Beta-2-glycoprotein I_Apo H.ATVVYQGER.S-120309-00025.2.y6.heavy	65	25	2
511.8	652.3		10	Beta-2-glycoprotein I_Apo H.ATVVYQGER.S-120309-00025.2.y5.light	65	25	2
511.8	751.4		10	Beta-2-glycoprotein I_Apo H.ATVVYQGER.S-120309-00025.2.y6.light	65	25	2
523.3	873.4	P02655	10	Apolipoprotein C-II.TAAQNLYEK.S-120214-00030.2.y7.heavy	71	24	1
523.3	674.35		10	Apolipoprotein C-II.TAAQNLYEK.S-120214-00030.2.y5.heavy	71	25	1
519.3	865.4		10	Apolipoprotein C-II.TAAQNLYEK.S-120214-00030.2.y7.light	71	24	1
519.3	666.35		10	Apolipoprotein C-II.TAAQNLYEK.S-120214-00030.2.y5.light	71	25	1
789.5	1075.6	P08519	10	Apolipoprotein(a).LFLEPTQADIALLK.S-091109-00015.2.y10.heavy	81	35	2
789.5	538.3		10	Apolipoprotein(a).LFLEPTQADIALLK.S-091109-00015.2.y++10.heavy	81	38	2
786.5	1069.6		10	Apolipoprotein(a).LFLEPTQADIALLK.S-091109-00015.2.y10.light	81	35	2
786.5	535.3		10	Apolipoprotein(a).LFLEPTQADIALLK.S-091109-00015.2.y++10.light	81	38	2
414.3	496.3	O95445	10	Apo M.AFLLTPR.S-110729-00002.2.y4.heavy	70	30	1
414.3	609.4		10	Apo M.AFLLTPR.S-110729-00002.2.y5.heavy	70	30	1
409.3	486.3		10	Apo M.AFLLTPR.S-110729-00002.2.y4.light	70	30	1
409.3	599.4		10	Apo M.AFLLTPR.S-110729-00002.2.y5.light	70	30	1
595.83	949.51	P27169	10	PON 1.IQNILTEEPK.S-090805-00030.2.y8.heavy	65	28	1
595.83	722.38		10	PON 1.IQNILTEEPK.S-090805-00030.2.y6.heavy	65	28	1
592.83	943.51		10	PON 1.IQNILTEEPK.S-090805-00030.2.y8.light	65	28	1
592.83	716.38		10	PON 1.IQNILTEEPK.S-090805-00030.2.y6.light	65	28	1
593.9	760.3	Q15848	10	Adiponectin.IFYNQQNHYDGSTGK.S-110708-00020.3.y++13.heavy	60	25	2
591.3	756.3		10	Adiponectin.IFYNQQNHYDGSTGK.S-110708-00020.3.y++13.light	60	25	2
567.8	833.4	P43652	10	Afamin.DADPDTFFAK.S-120309-00027.2.y7.heavy	60	24	2
563.8	825.4		10	Afamin.DADPDTFFAK.S-120309-00027.2.y7.light	60	24	2
579.3	945.5	P02768	10	Albumin.LVNEVTEFAK.S-110708-00016.2.y8.heavy	70	25	2
575.3	937.5		10	Albumin.LVNEVTEFAK.S-110708-00016.2.y8.light	70	25	2
535.3	827.5	P01011	10	Alpha-1-antichymotrypsin.EIGELYLPK.S-120309-00023.2.y7.heavy	60	24	2
531.3	819.5		10	Alpha-1-antichymotrypsin.EIGELYLPK.S-120309-00023.2.y7.light	60	24	2
826.4	719.4	P01009	10	Alpha-1-Anti-trypsin.ITPNLAEFAFSLYR.BOR6121A01.2.y++12.heavy	95	34	2
821.4	714.4		10	Alpha-1-Anti-trypsin.ITPNLAEFAFSLYR.BOR6121A01.2.y++12.light	95	34	2
559.8	805.4		10	Alpha-1-Anti-trypsin.LSITGTYDLK.S-110124-00040.2.y7.heavy	56	27	2
555.8	797.4		10	Alpha-1-Anti-trypsin.LSITGTYDLK.S-110124-00040.2.y7.light	56	27	2
623.3	902.5	P04217	10	Alpha-1B-glycoprotein.LETPDFQLFK.S-120309-00016.2.y7.heavy	65	28	2
619.3	894.5		10	Alpha-1B-glycoprotein.LETPDFQLFK.S-120309-00016.2.y7.light	65	28	2
660.85	779.4	P08697	10	Alpha-2-antiplasmin.LGNQEPGGQTALK.S-110708-00012.2.y8.heavy	82	33	2
656.85	771.4		10	Alpha-2-antiplasmin.LGNQEPGGQTALK.S-110708-00012.2.y8.light	82	33	2
364.2	348.2	P02765	10	Alpha-2-HS-glycoprotein.APHGPGLIYR.BOR6121A04.3.y2.heavy	40	20	2
360.9	338.2		10	Alpha-2-HS-glycoprotein.APHGPGLIYR.BOR6121A04.3.y2.light	40	20	2
402.2	498.3		10	Alpha-2-HS-glycoprotein.HTLNQIDEVK.S-110124-00041.3.y4.heavy	45	17	2
399.5	490.3		10	Alpha-2-HS-glycoprotein.HTLNQIDEVK.S-110124-00041.3.y4.light	45	17	2

561	869.5	P01023	10	Alpha-2-macroglobulin.TEHPFTVEEFVLPK.3.BOR6121A05.y7.heavy	57	23	2
558.3	861.5		10	Alpha-2-macroglobulin.TEHPFTVEEFVLPK.3.BOR6121A05.y7.light	57	23	2
638.9	546.8	P01019	10	Angiotensinogen.ALQDQLVLVAAK.S-120309-00028.2.y++10.heavy	73	25	2
634.9	542.8		10	Angiotensinogen.ALQDQLVLVAAK.S-120309-00028.2.y++10.light	73	25	2
439.9	544.3	P01008	10	Antithrombin-III.DDLYVSDAFHK.S-110708-00009.3.y++9.heavy	47	17	2
437.2	540.3		10	Antithrombin-III.DDLYVSDAFHK.S-110708-00009.3.y++9.light	47	17	2
649.8	812.4	P10909	10	Clusterin.ELDESLQVAER.S-120309-00017.2.y7.heavy	75	33	2
644.8	802.4		10	Clusterin.ELDESLQVAER.S-120309-00017.2.y7.light	75	33	2
649.8	612.3		10	Clusterin.ELDESLQVAER.S-120309-00017.2.y5.heavy	75	31	2
644.8	602.3		10	Clusterin.ELDESLQVAER.S-120309-00017.2.y5.light	75	31	2
563.8	585.3	P05155	10	Complement C1 inactivator.LLDSLPSDTR.HPP-0107.2.y5.heavy	65	25	2
558.8	575.3		10	Complement C1 inactivator.LLDSLPSDTR.HPP-0107.2.y5.light	65	25	2
505.8	739.4	P01024	10	Complement C3.TGLQEVEVK.S-110708-00007.2.y6.heavy	65	25	2
501.8	731.4		10	Complement C3.TGLQEVEVK.S-110708-00007.2.y6.light	65	25	2
562.8	639.4	P0C0L5	10	Complement C4 beta chain.VDGTLNLNLR.S-110708-00010.2.y5.heavy	70	31	2
557.8	629.4		10	Complement C4 beta chain.VDGTLNLNLR.S-110708-00010.2.y5.light	70	31	2
365.5	491.3	P0C0L5	10	Complement C4 gamma chain.ITQVLHFTK.S-120309-00020.3.y++8.heavy	88	16	2
362.9	487.3		10	Complement C4 gamma chain.ITQVLHFTK.S-120309-00020.3.y++8.light	88	16	2
511.2	502.3	P02748	10	Complement C9.TEHYEEQIEAFK.S-120309-00030.3.y4.heavy	66	23	2
508.6	494.3		10	Complement C9.TEHYEEQIEAFK.S-120309-00030.3.y4.light	66	23	2
625.9	525.8	P02748	10	Complement C9.LSPIYNLVPVK.S-100225-00108.2.y++9.heavy	54	26	2
621.9	521.8		10	Complement C9.LSPIYNLVPVK.S-100225-00108.2.y++9.light	54	26	2
582.3	679.4	P00751	10	Complement factor B.EELLPAQDIK.S-120309-00001.2.y6.heavy	65	25	2
578.3	671.4		10	Complement factor B.EELLPAQDIK.S-120309-00001.2.y6.light	65	25	2
675.4	838.4	P08603	10	Complement factor H.SPDVINGSPISQK.S-120309-00009.2.y8.heavy	77	35	2
671.4	830.4		10	Complement factor H.SPDVINGSPISQK.S-120309-00009.2.y8.light	77	35	2
574.8	875.5	P02671	10	Fibrinogen alpha chain.GSESGIFTNTK.S-120309-00036.2.y8.heavy	61	28	2
570.8	867.5		10	Fibrinogen alpha chain.GSESGIFTNTK.S-120309-00036.2.y8.light	61	28	2
658.8	714.3	P02675	10	Fibrinogen beta chain.QGFGNVATNTDGK.S-120309-00026.2.y7.heavy	85	34	2
654.8	706.3		10	Fibrinogen beta chain.QGFGNVATNTDGK.S-120309-00026.2.y7.light	85	34	2
501.3	605.3	P02679	10	Fibrinogen gamma chain.YEASILTHDSSIR.HPP-0076.3.y++11.heavy	61	21	2
497.9	600.3		10	Fibrinogen gamma chain.YEASILTHDSSIR.HPP-0076.3.y++11.light	61	21	2
773.8	1087.5	P02671	10	Fibrinopeptide A.ADSGEGDFLAEGGGVR.HPP-0099.2.y11.heavy	66	37	2
768.8	1077.5		10	Fibrinopeptide A.ADSGEGDFLAEGGGVR.HPP-0099.2.y11.light	66	37	2
495.8	572.3	P00738	10	Haptoglobin beta chain.VGYVSGWGR.S-120309-00007.2.y5.heavy	61	25	2
490.8	562.3		10	Haptoglobin beta chain.VGYVSGWGR.S-120309-00007.2.y5.light	61	25	2
615.8	485.3	P02790	10	Hemopexin.NFPSPVDAAFR.S-110708-00005.2.y++9.heavy	59	25	2
610.8	480.3		10	Hemopexin.NFPSPVDAAFR.S-110708-00005.2.y++9.light	59	25	2
519.8	824.4	P05546	10	Heparin cofactor II.TLEAQLTPR.S-120309-00018.2.y7.heavy	65	25	2
514.8	814.4		10	Heparin cofactor II.TLEAQLTPR.S-120309-00018.2.y7.light	65	25	2
584.3	912.5	P19827	10	Inter-alpha-trypsin inhibitor HC.AAISGENAGLVR.S-120309-00031.2.y9.heavy	66	28	2

579.3	902.5		10	Inter-alpha-trypsin inhibitor HC.AAISGENAGLVR.S-120309-00031.2.y9.light	66	28	2
630.3	1059.5	P01042	10	Kininogen-1.TVGSDTFYSFK.S-120309-00021.2.y9.heavy	62	26	2
626.3	1051.5		10	Kininogen-1.TVGSDTFYSFK.S-120309-00021.2.y9.light	62	26	2
501.8	802.4	P14151	10	L-selectin.AEIEYLEK.S-110708-00014.2.y6.heavy	64	21	2
497.8	794.4		10	L-selectin.AEIEYLEK.S-110708-00014.2.y6.light	64	21	2
603.8	701.4	P02753	10	Plasma retinol-binding protein.YWGVASFLQK.S-120309-00019.2.y6.heavy	76	29	2
599.8	693.4		10	Plasma retinol-binding protein.YWGVASFLQK.S-120309-00019.2.y6.light	76	29	2
630.3	687.4	P00734	10	Prothrombin.ETAASLLQAGYK.S-120309-00033.2.y6.heavy	70	29	2
626.3	679.4		10	Prothrombin.ETAASLLQAGYK.S-120309-00033.2.y6.light	70	29	2
583.8	718.4	P02743	10	Serum amyloid P-component.VGEYSLYIGR.S-120309-00010.2.y6.heavy	80	27	2
578.8	708.4		10	Serum amyloid P-component.VGEYSLYIGR.S-120309-00010.2.y6.light	80	27	2
647.3	781.4	P02787	10	Transferrin.EGYYGYTGAFR.S-110124-00050.2.y7.heavy	73	31	2
642.3	771.4		10	Transferrin.EGYYGYTGAFR.S-110124-00050.2.y7.light	73	31	2
701.8	614.3	P02766	10	Transthyretin.AADDTWEPFASGK.S-120309-00012.2.y6.heavy	70	38	2
697.8	606.3		10	Transthyretin.AADDTWEPFASGK.S-120309-00012.2.y6.light	70	38	2
393.6	355.2	P02774	10	Vitamin D-binding protein.THLPEVFLSK.S-120309-00003.3.y3.heavy	44	18	2
390.9	347.2		10	Vitamin D-binding protein.THLPEVFLSK.S-120309-00003.3.y3.light	44	18	2
623.8	1020.5	P04275	10	von Willebrand Factor.ILAGPAGDSNVVK.HPP-0108.2.y11.heavy	75	29	2
620.9	1014.5		10	von Willebrand Factor.ILAGPAGDSNVVK.HPP-0108.2.y11.light	75	29	2
896	774.9	P25311	10	Zinc-alpha-2-glycoprotein.EIPAWVPFDPAAQITK.S-120309-00004.2.y++14.heavy	55	35	2
892	770.9		10	Zinc-alpha-2-glycoprotein.EIPAWVPFDPAAQITK.S-120309-00004.2.y++14.light	55	35	2
896	1095.6		10	Zinc-alpha-2-glycoprotein.EIPAWVPFDPAAQITK.S-120309-00004.2.y10.heavy	55	40	2
892	1087.6		10	Zinc-alpha-2-glycoprotein.EIPAWVPFDPAAQITK.S-120309-00004.2.y10.light	55	40	2

MRM parameters on proteotypic tryptic peptides for 56 plasma proteins used to screen for HDL proteins.

Method 1 refers to new transitions developed using tryptic digestion of recombinant proteins as described in the method supplement and in (Yassine et al., 2013).

Method 2 refers to transitions were previously published (Kuzyk et al., 2009)

Figure S1 :Representative Chromatograms of HDL peptides by MRM

Chromatograms representative of several HDL peptides from the control sample pool are shown. Two transitions per peptide were used. The 4 replicate runs are presented.

Apo M. AFLLTPR.S-110729-00002.y5

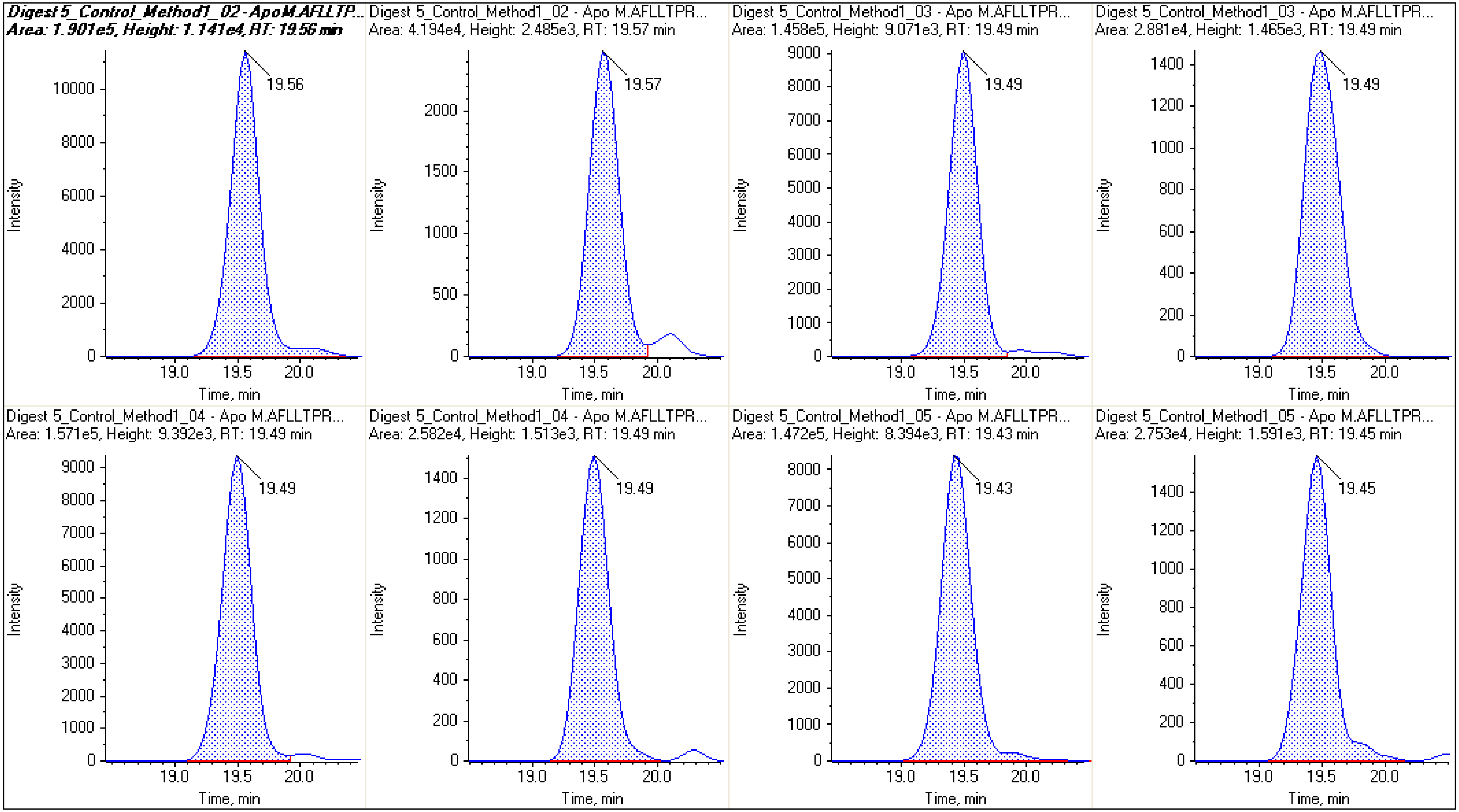

Apolipoprotein A-1. ATEHLSTLSEK.BOR5409A9.1

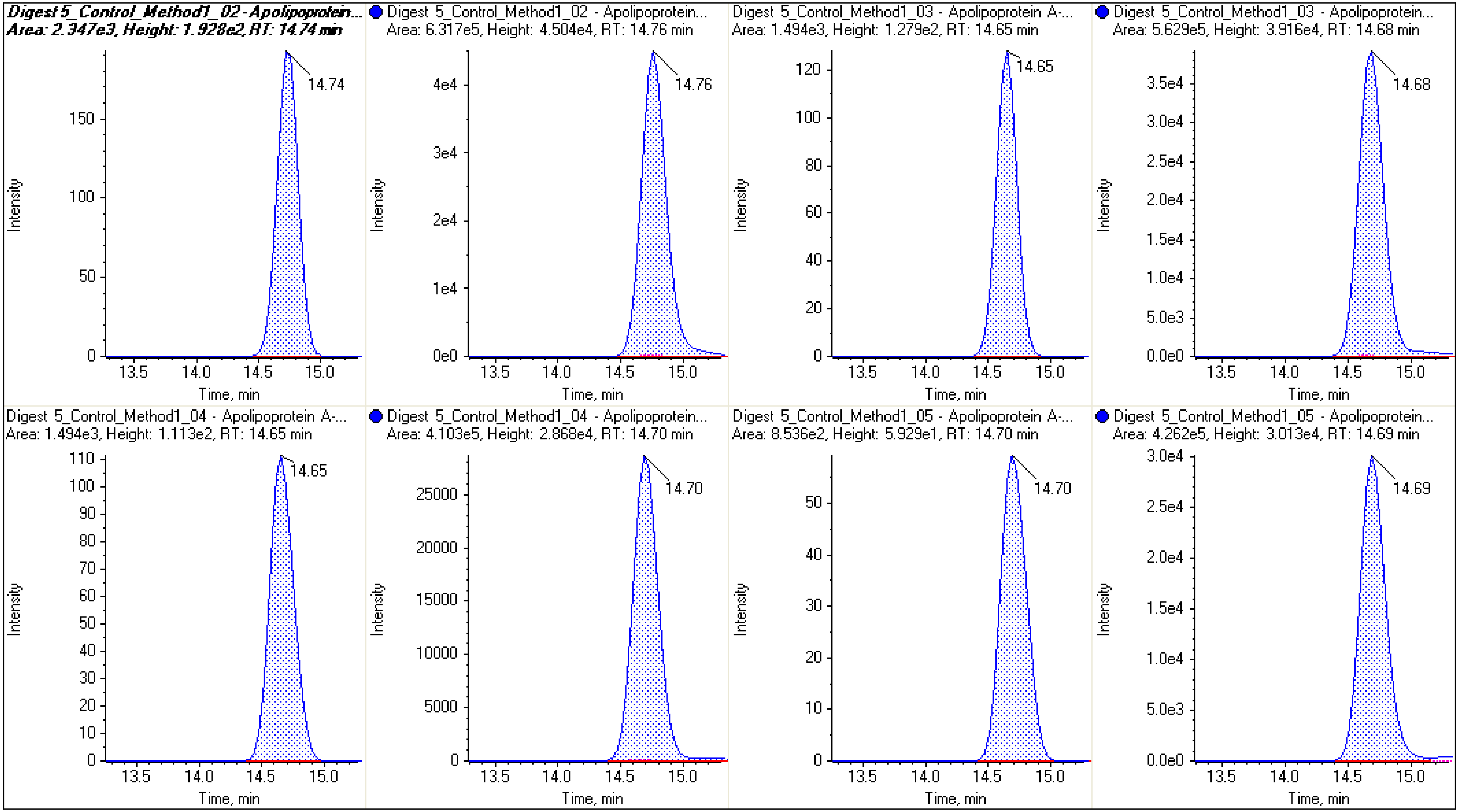

Apolipoprotein C-II.TAAQNLYEK.S-090813-00004.y5

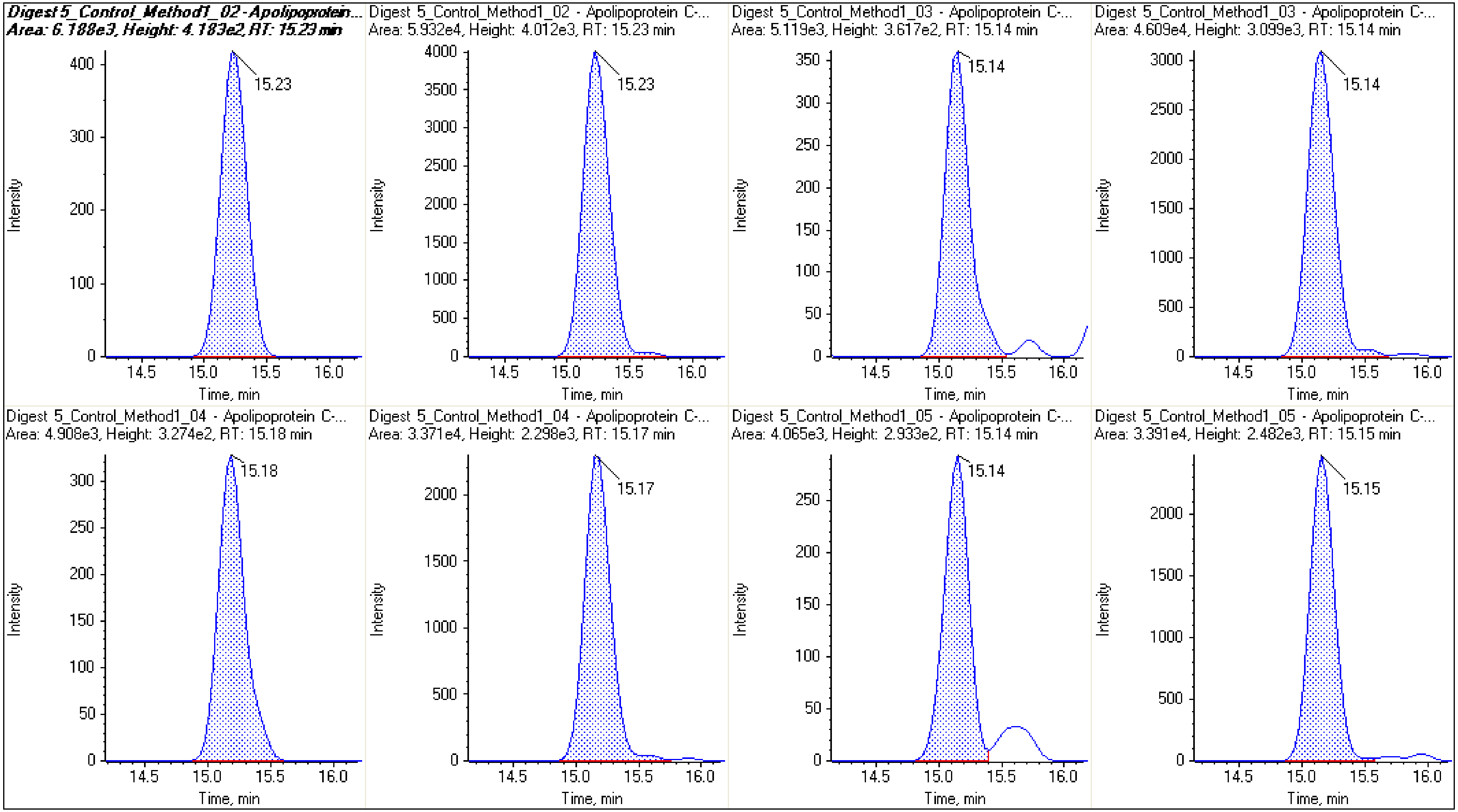

Apolipoprotein C-III.GWVTDGFSSLK.BOR5775A1.1

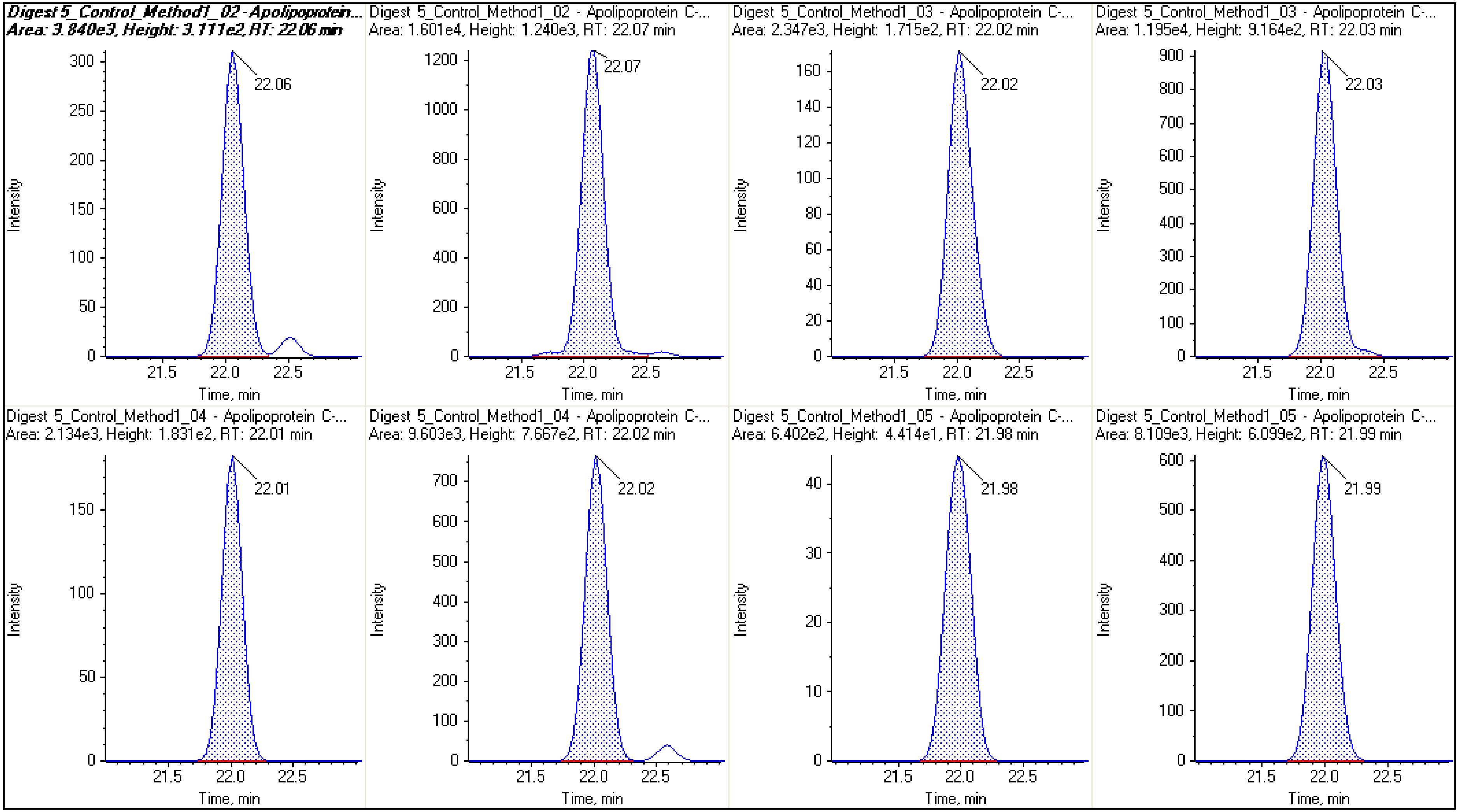

Apolipoprotein C-III.GWVTDGFSSLK.BOR5775A1.2

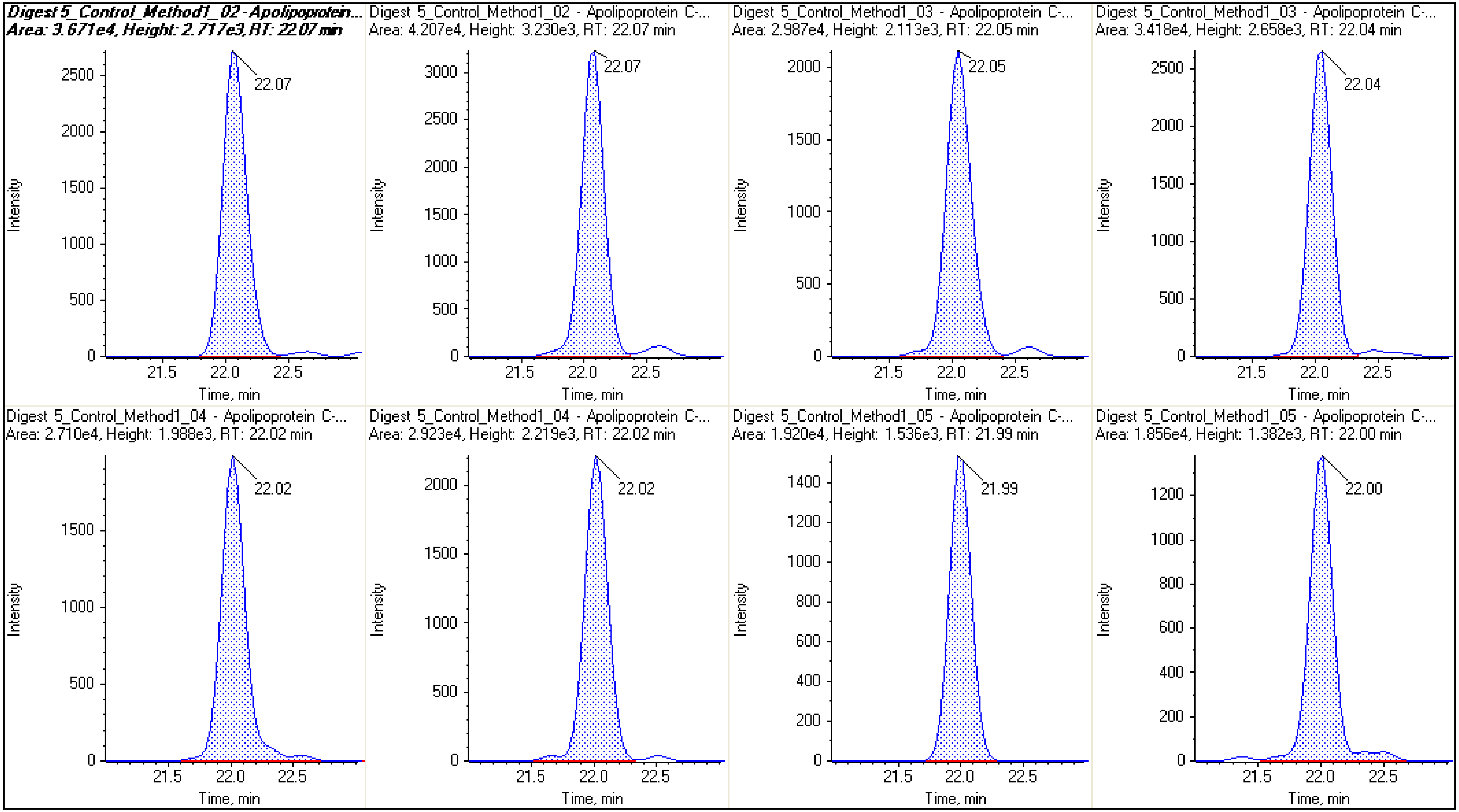

Apolipoprotein C-III.GWVTDGFSSLK.BOR5775A1.3

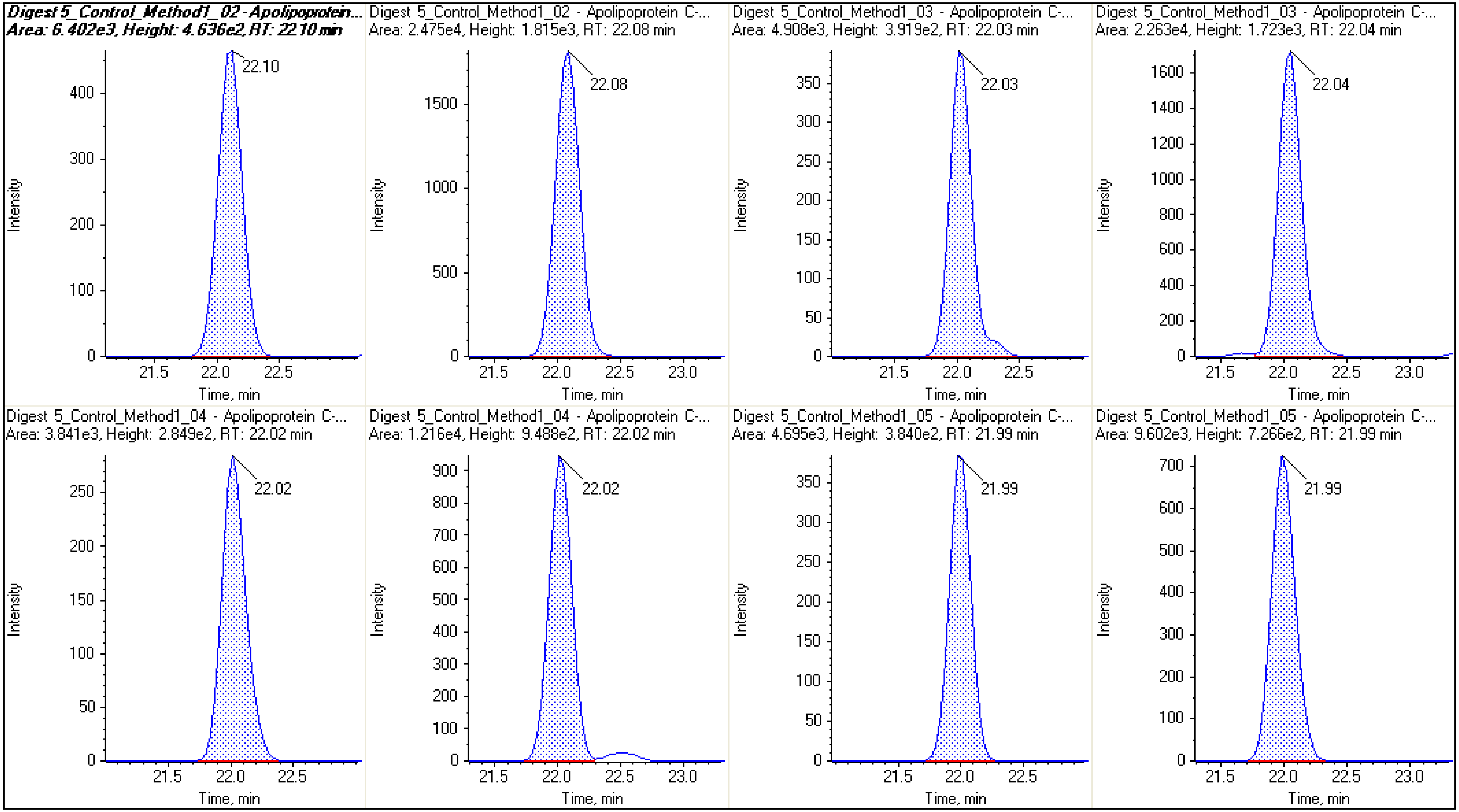

Apolipoprotein D.VLNQELR.S-090813-00010.y++6

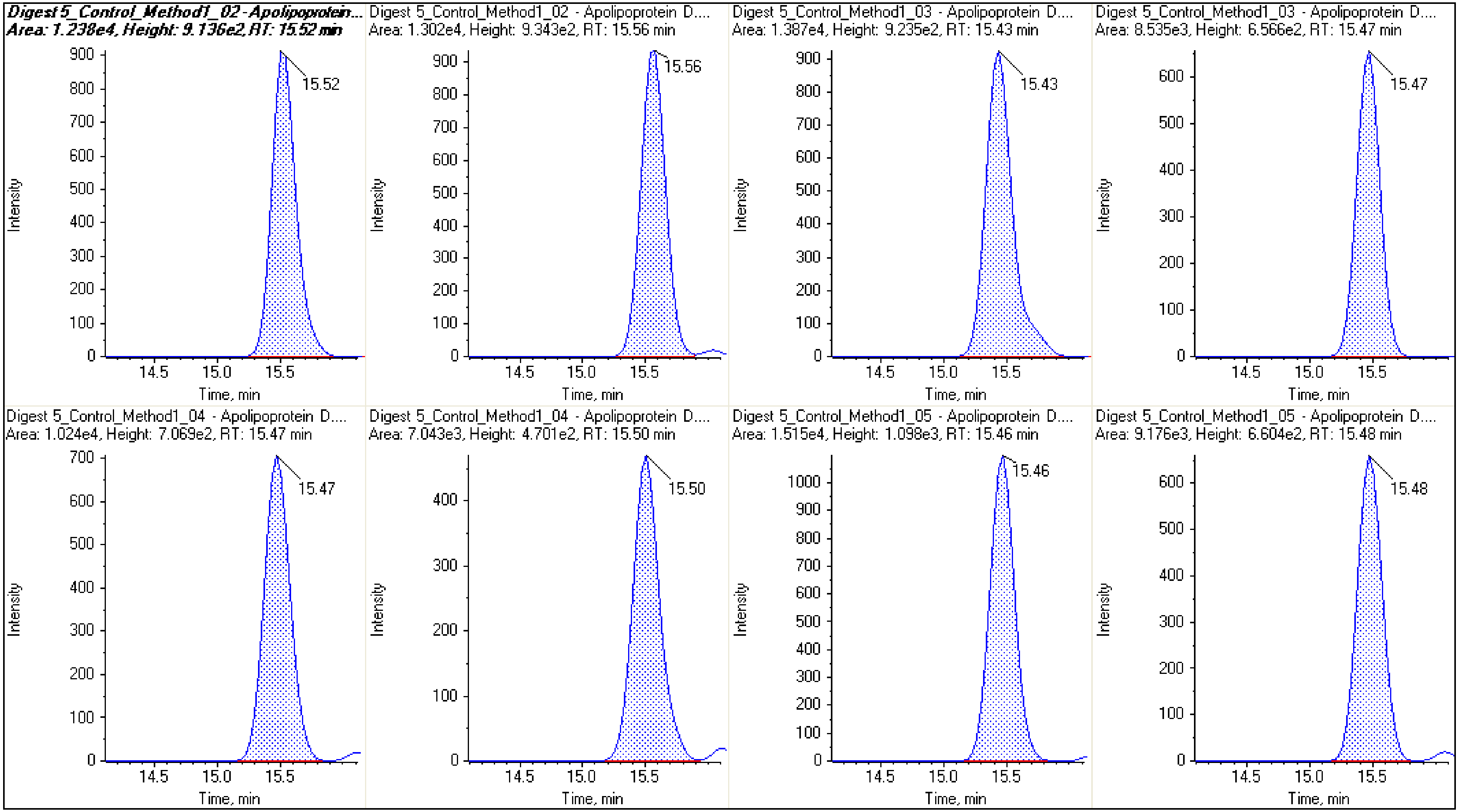

Apolipoprotein(a).LFLEPTQADIALLK.S-091109-00015.y++10

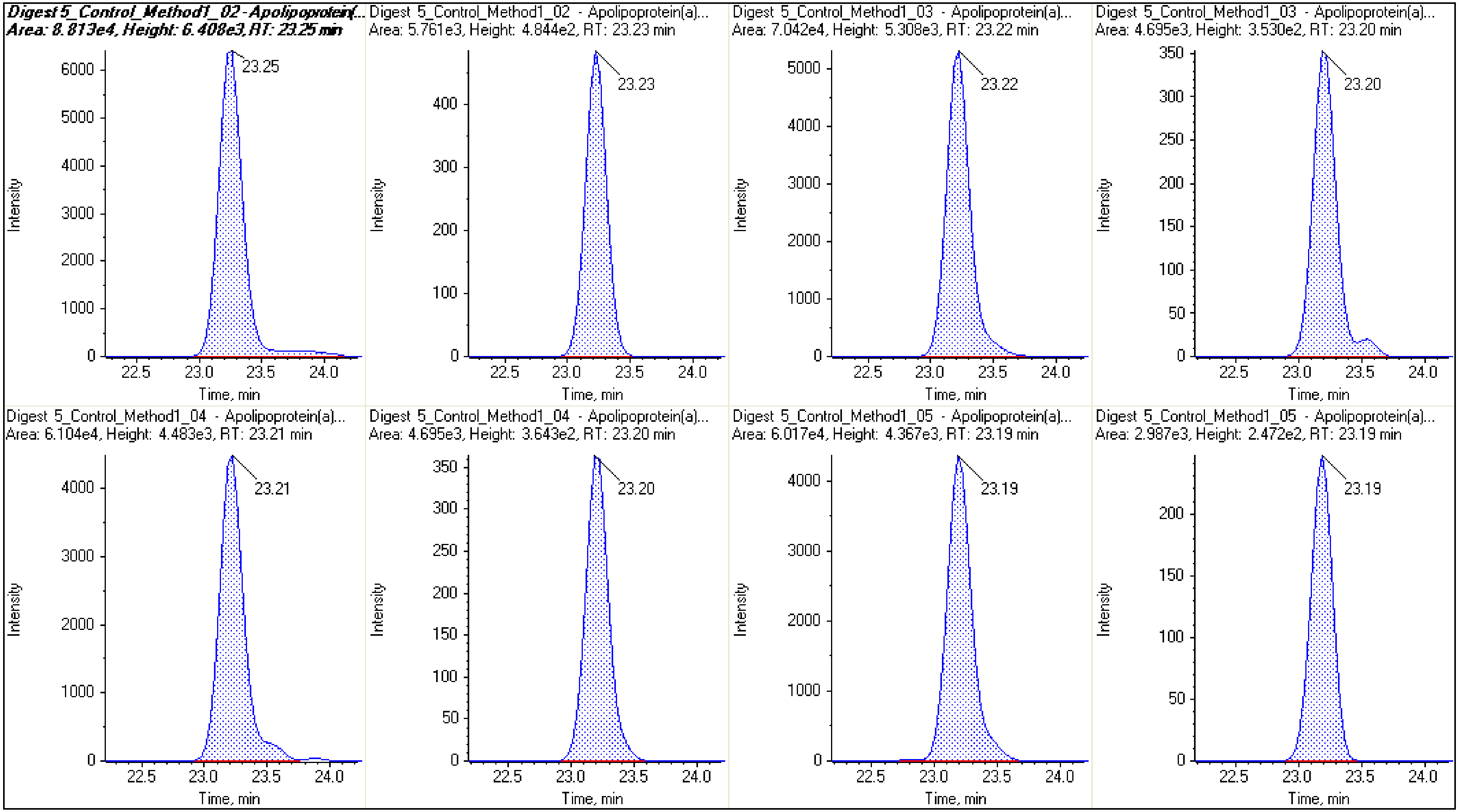

Apolipoprotein(a).LFLEPTQADIALLK.S-091109-00015.y10

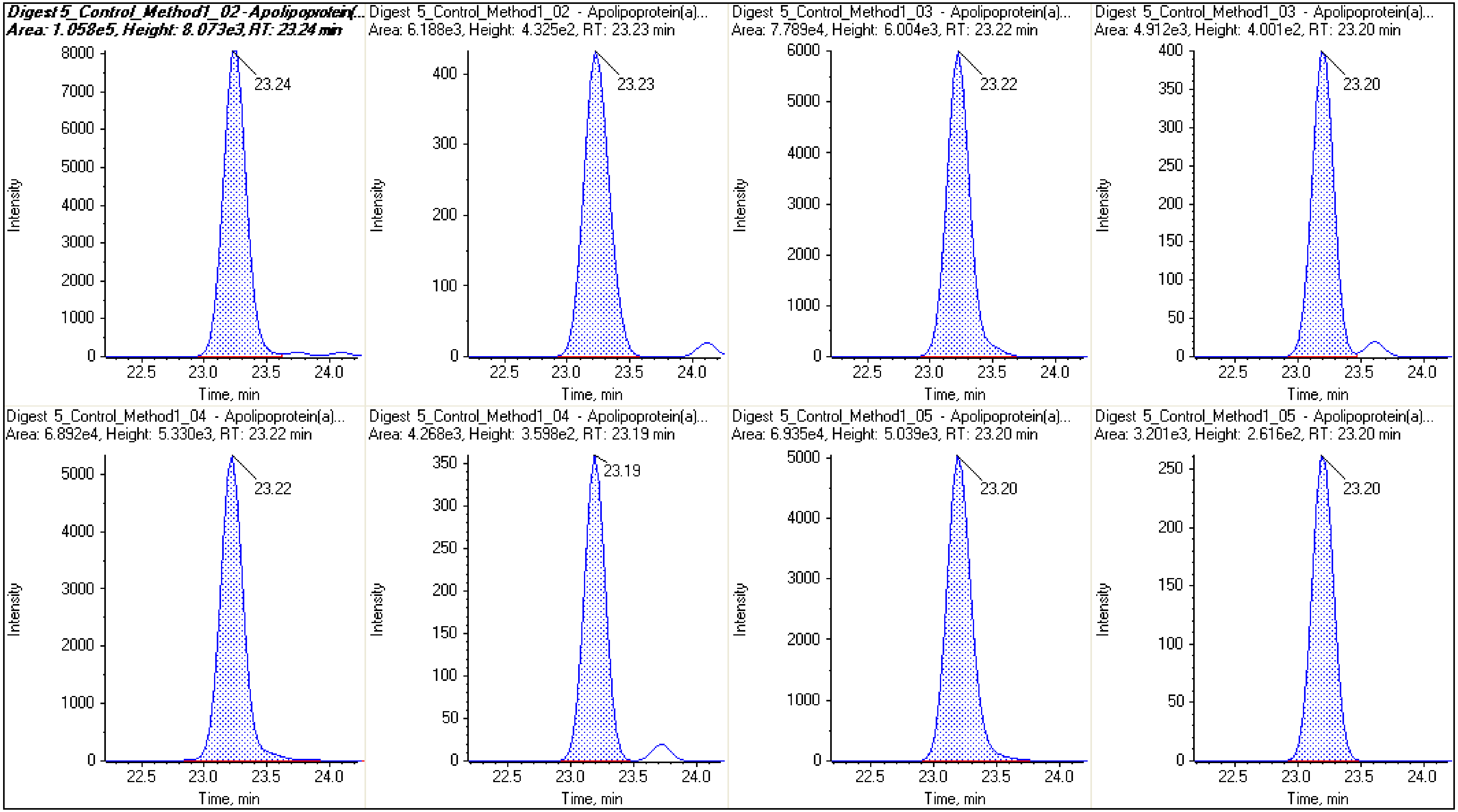

Human Matrix Gla.NANTFISPQQR.S-120215-00006.y5

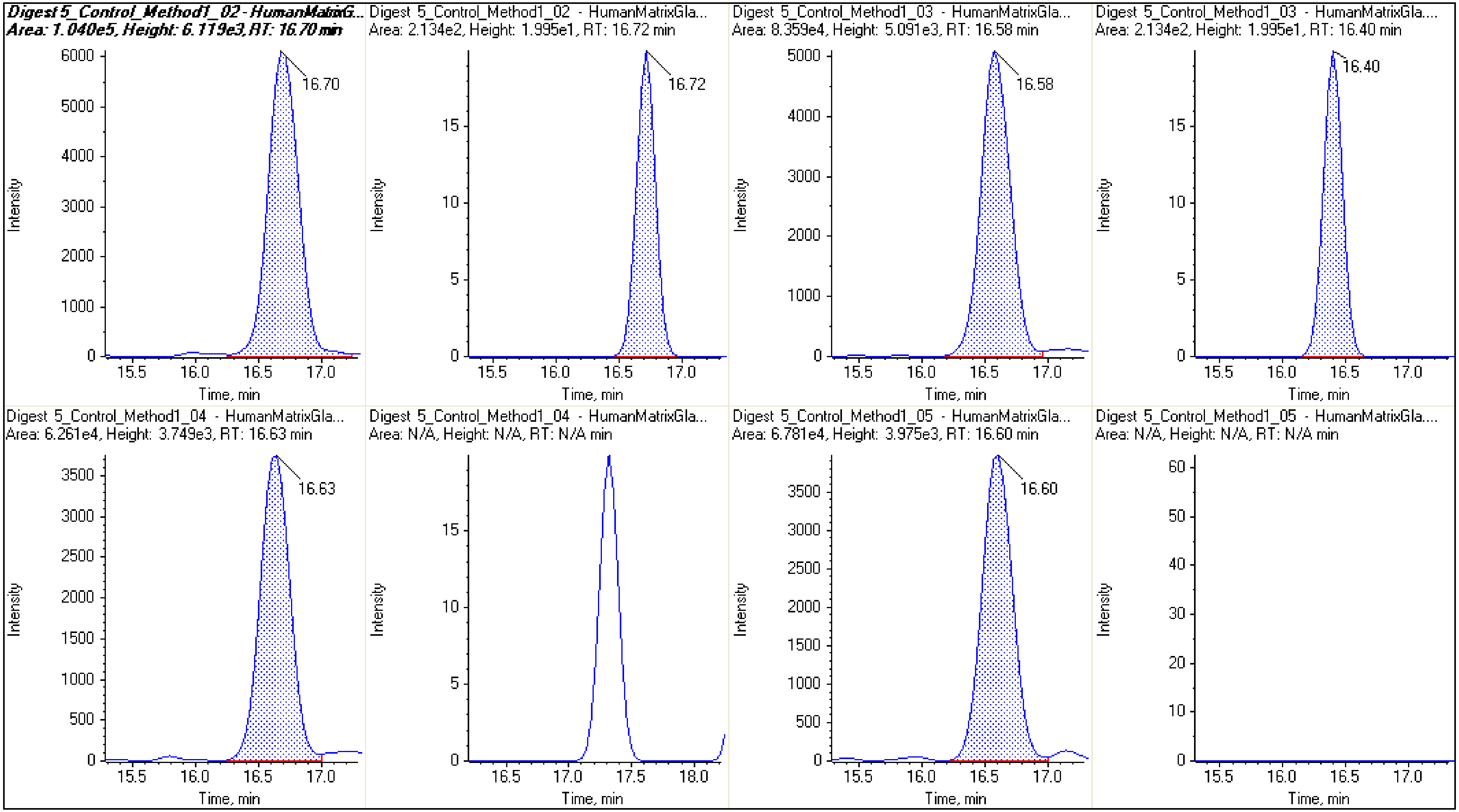

Macrophage Migration Inhib Factor.LLCGLLAER.S-120215-00008.y7

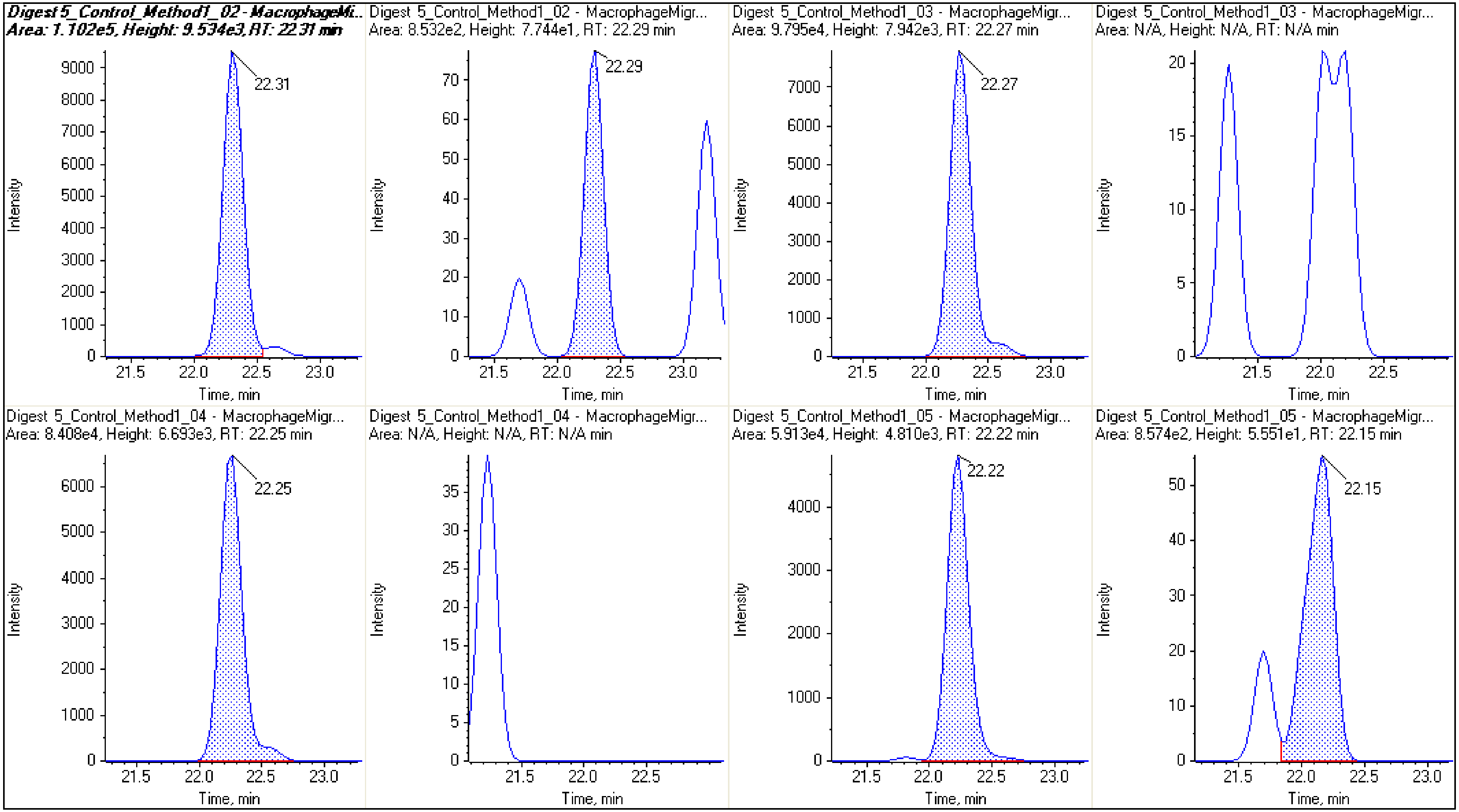

PON 1.IQNILTEEPK.S-090805-00030.2y8.2

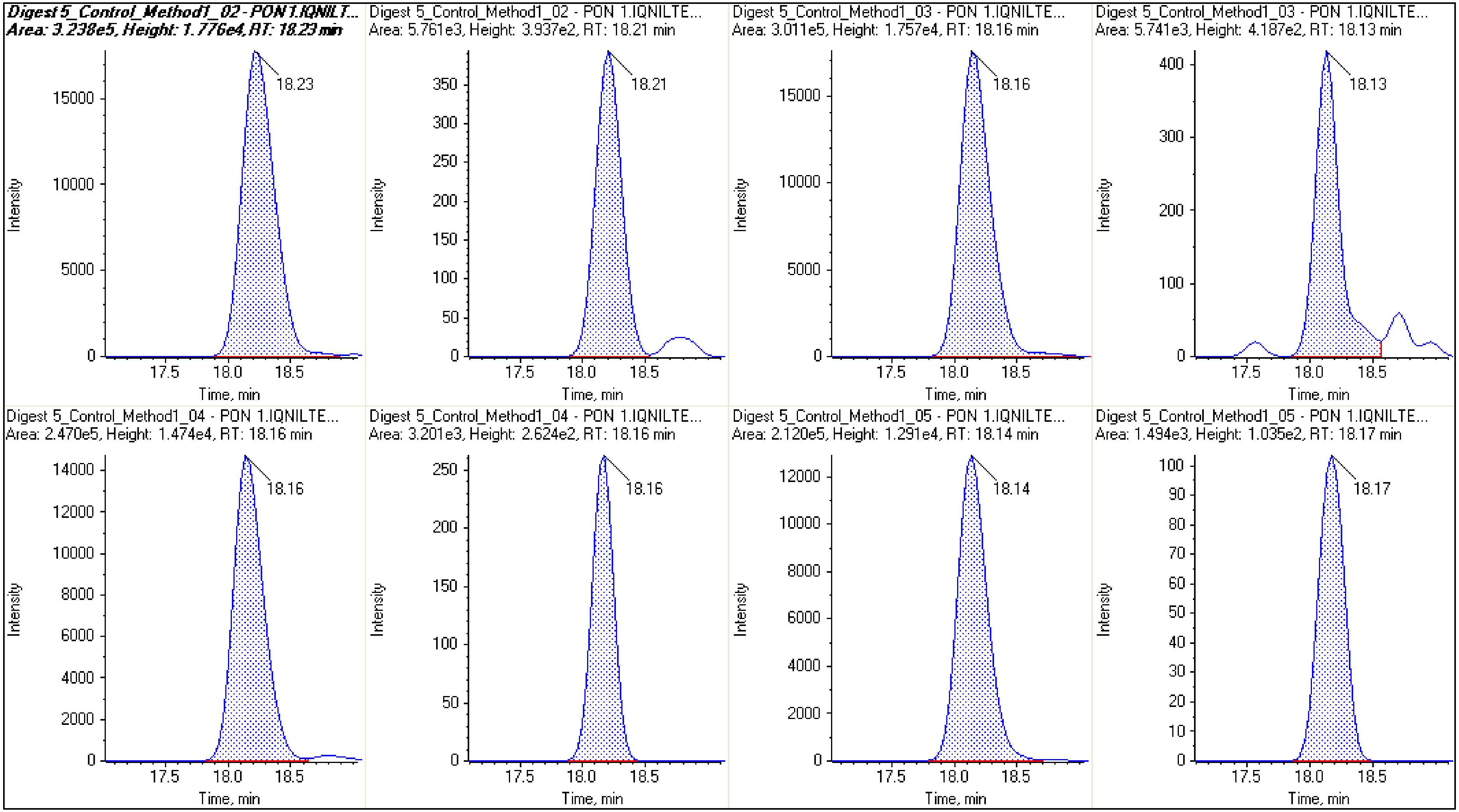

Albumin.LVNEVTEFAK.BOR5409A01.y8

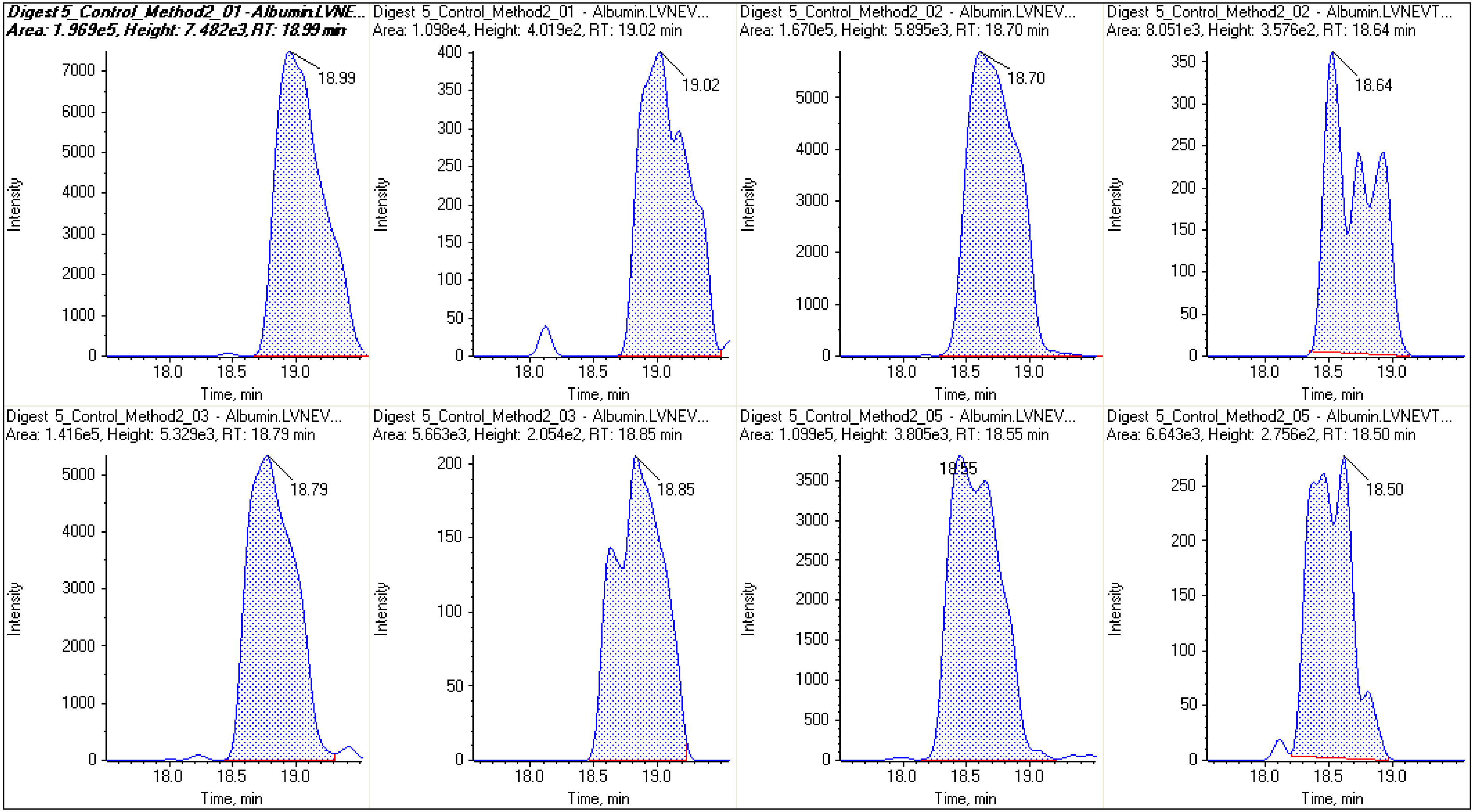

Apo M.AFLLTPR.S-110729-00002.y4

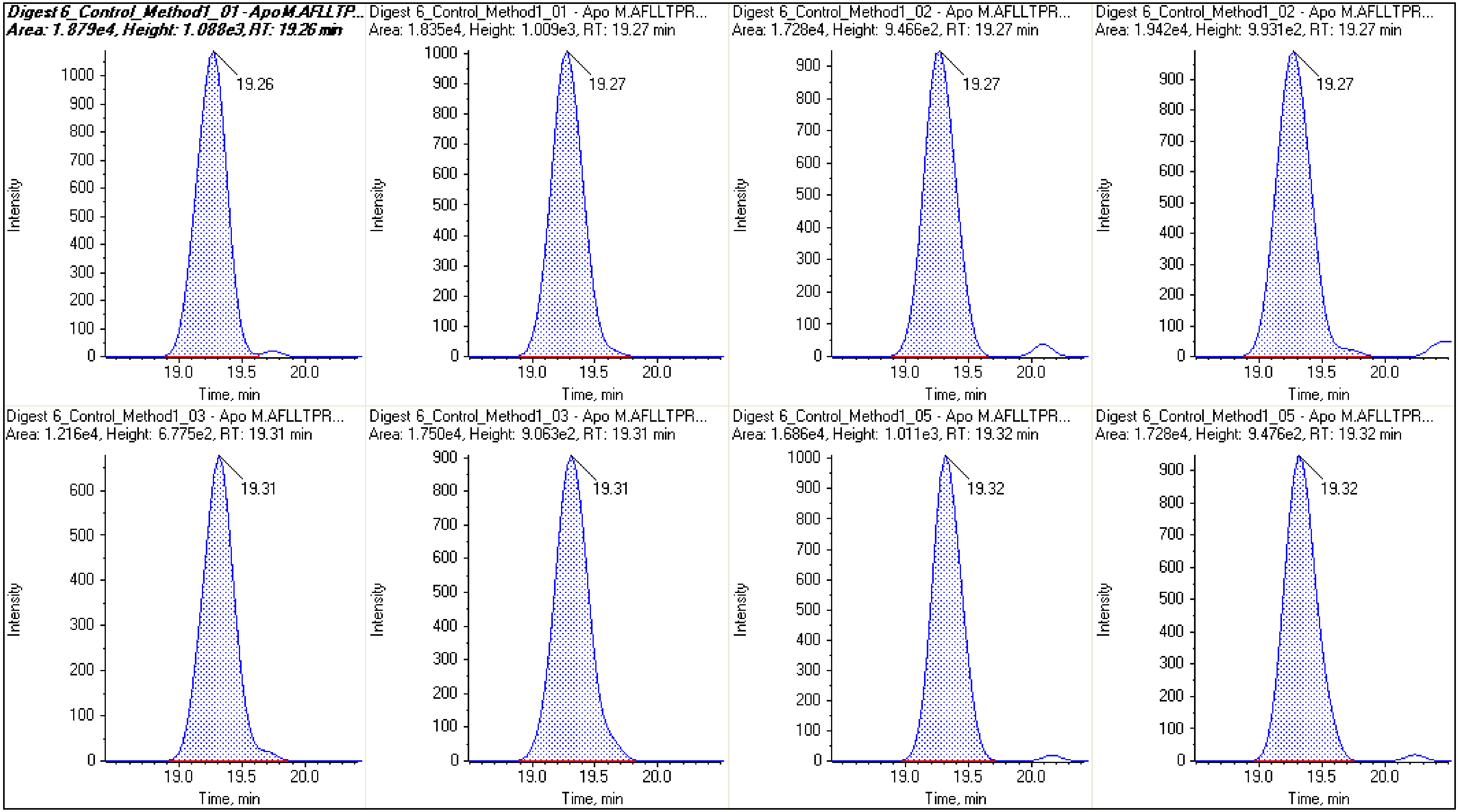

Apo M.AFLLTPR.S-110729-00002.y5

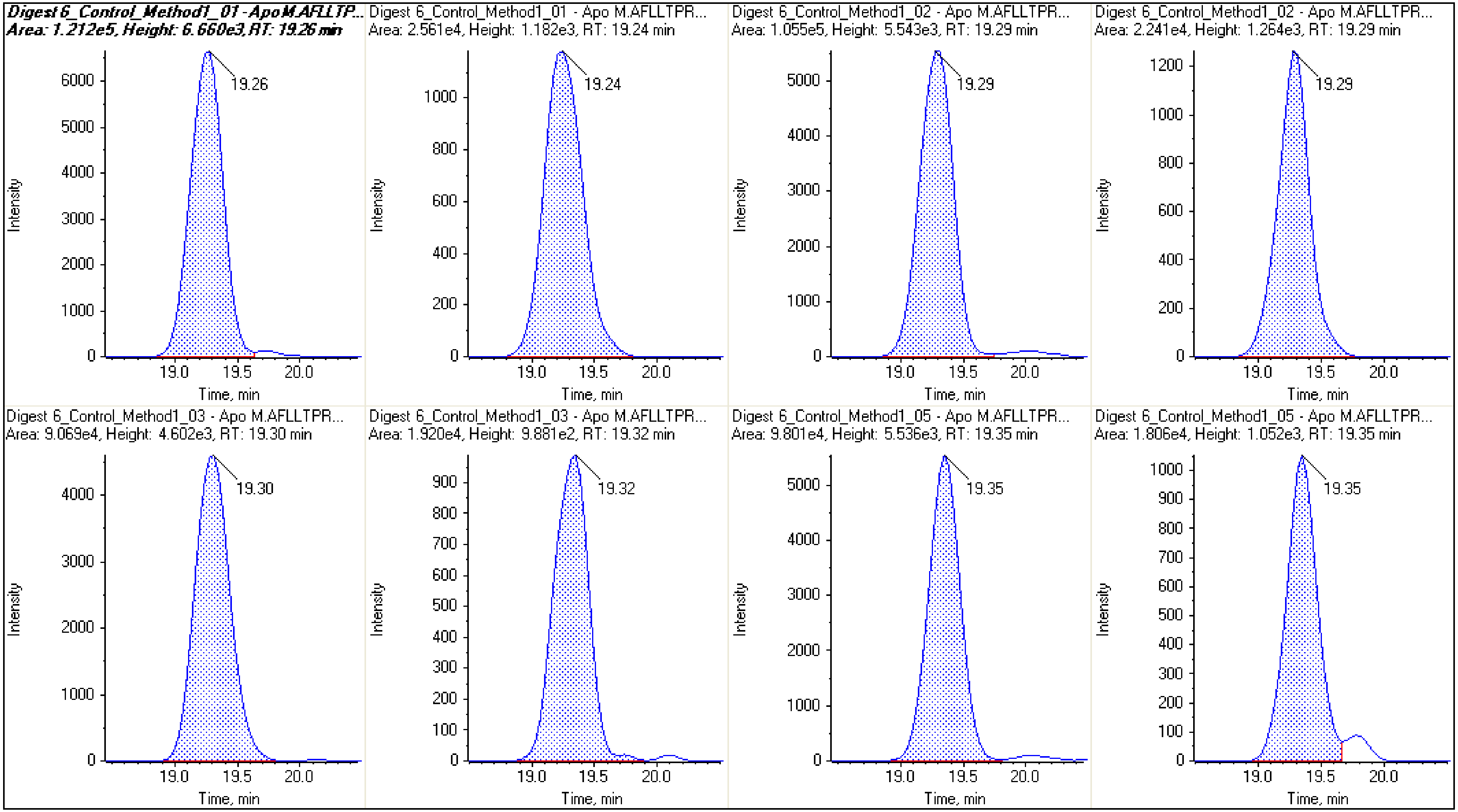

Apolipoprotein A-II precursor.SPELQAEAK.BOR5409A12.1

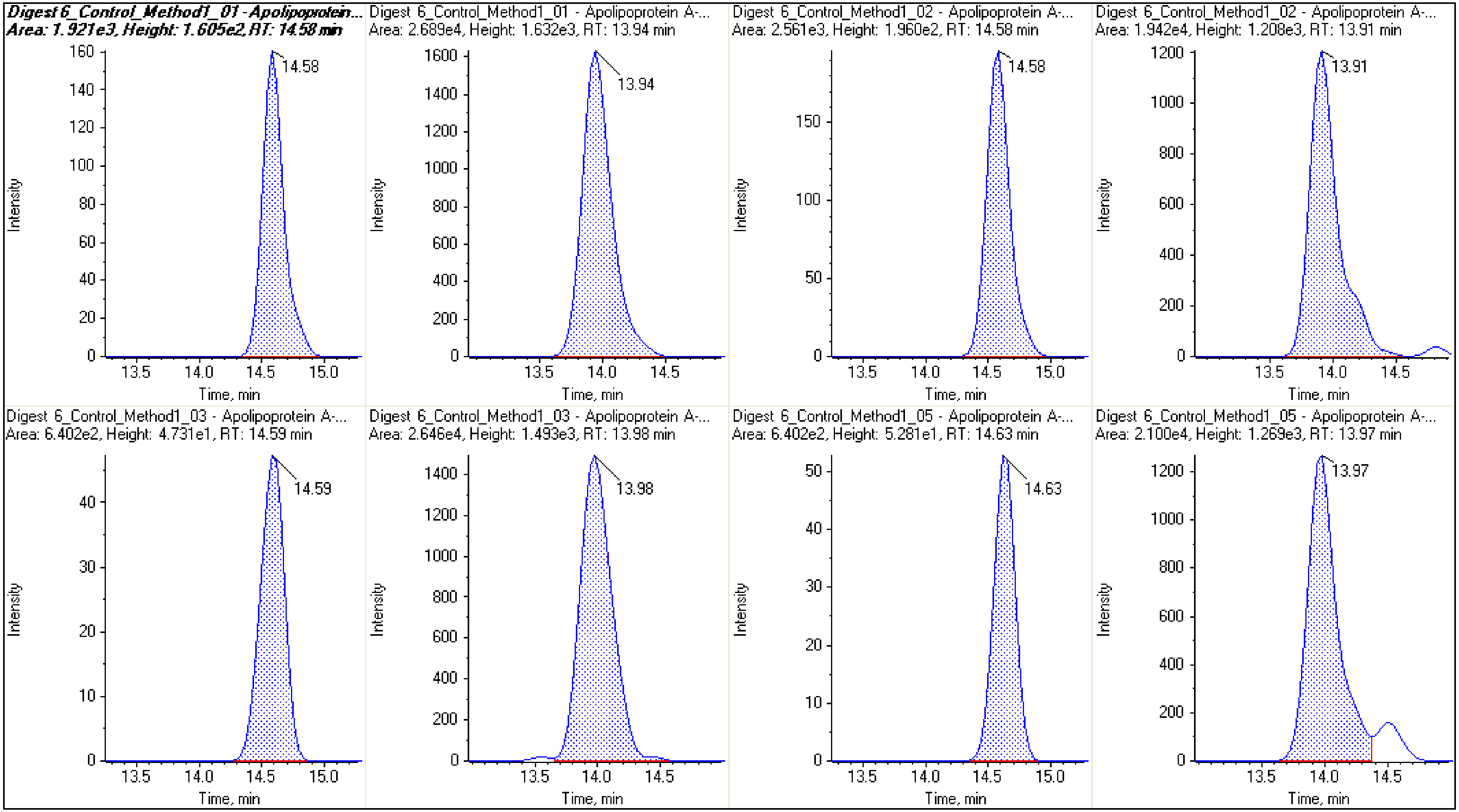

Apolipoprotein C-II.TAAQNLYEK.S-090813-00004.y5

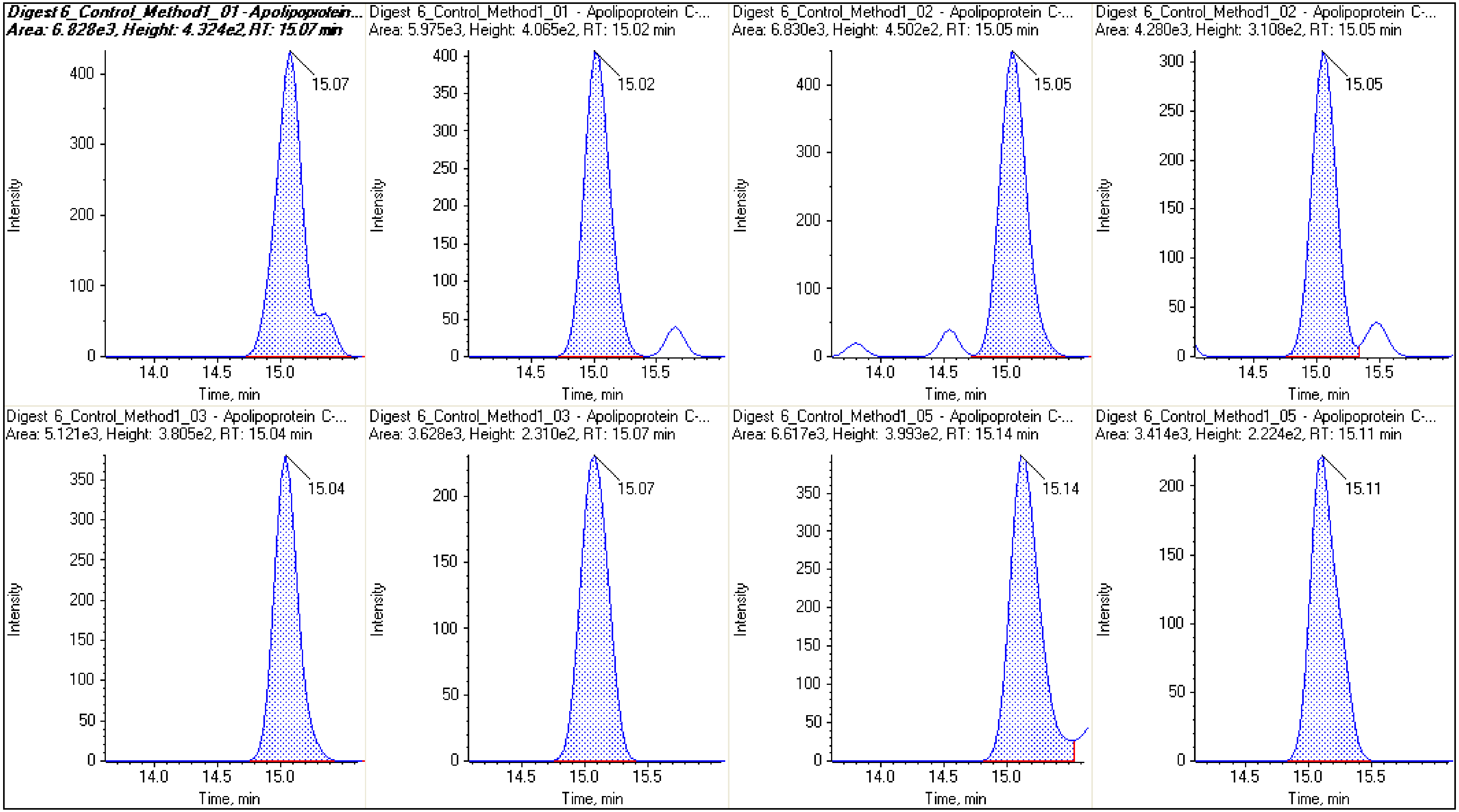

Apolipoprotein C-II.TAAQNLYEK.S-090813-00004.y7

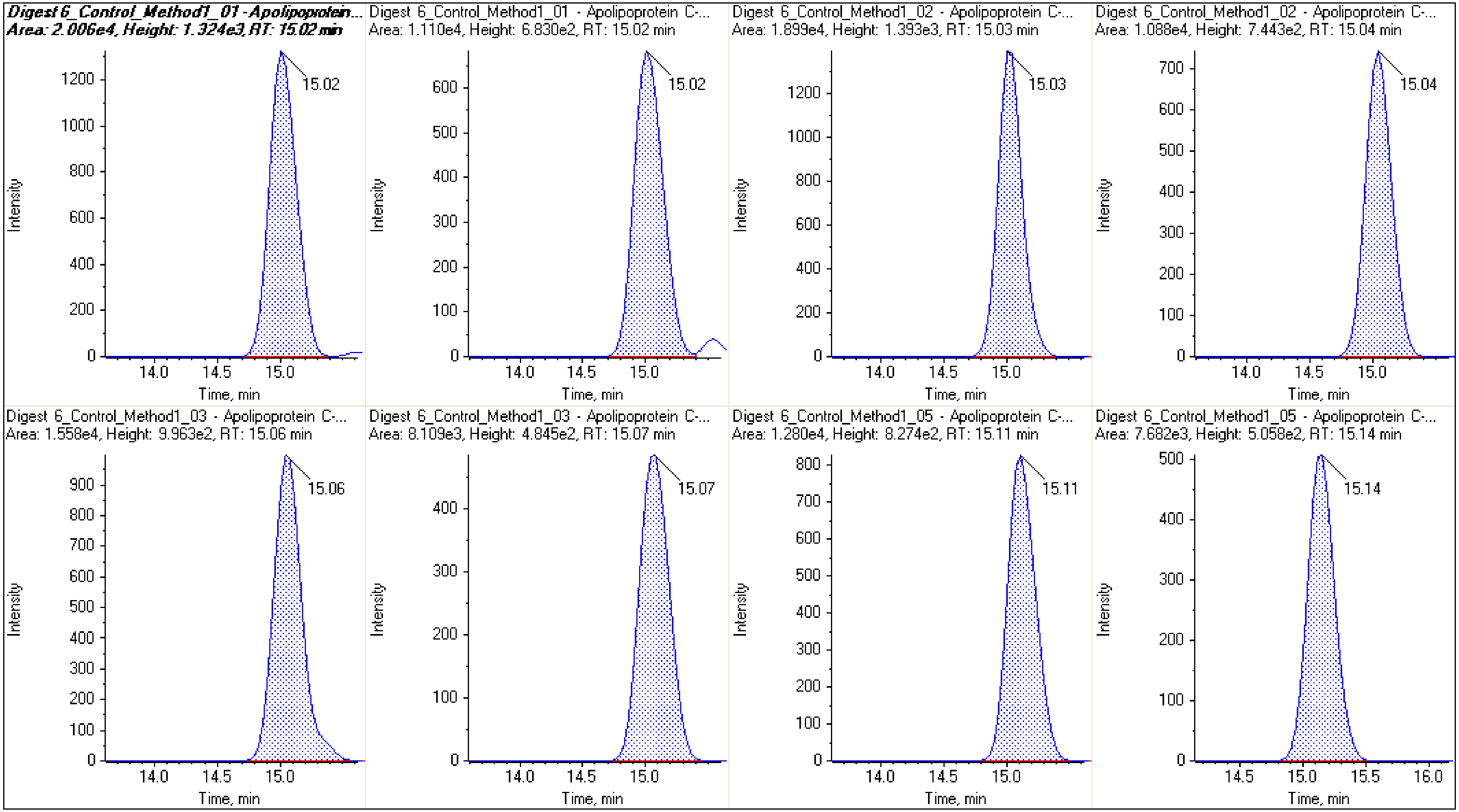

Albumin.LVNEVTEFAK.BOR5409A1.y8

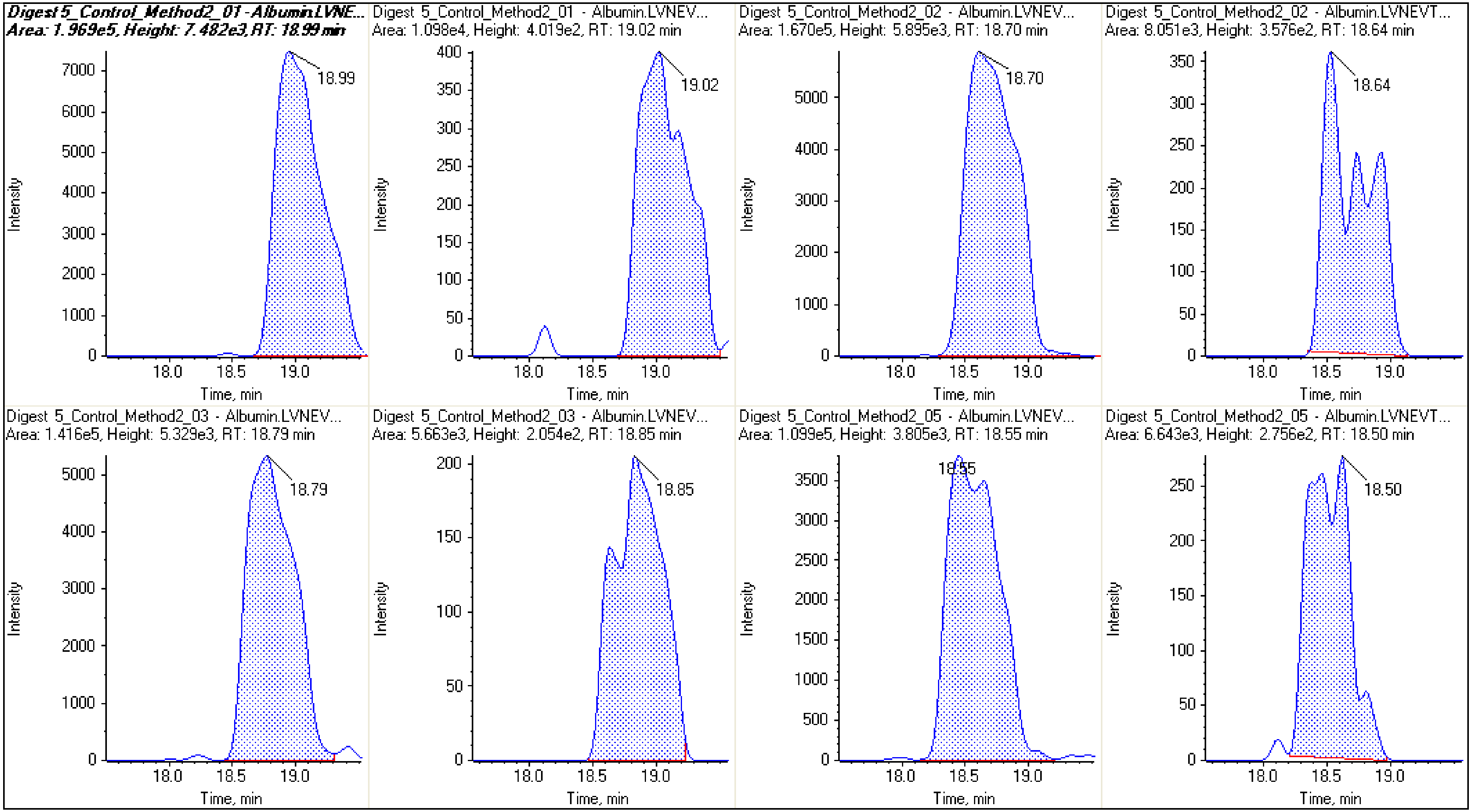

Supplement: Additional file 1 — lists (1) details of MRM method summary. (2) Table S1. showing the HDL protein concentrations after 12 dilutions for determination of lower limit of detection (linearity analysis) (3) Table S2. listing all the transitions used to screen for HDL proteins (4) Figure S1. showing representative chromatograms from the 4 replicate runs. [file 1476-511X-13-8-S1.pdf]
